# Lineage tracing in the adult mouse corneal epithelium supports the limbal epithelial stem cell hypothesis with intermittent periods of stem cell quiescence^☆^

**DOI:** 10.1016/j.scr.2015.10.016

**Published:** 2015-11

**Authors:** Natalie J. Dorà, Robert E. Hill, J. Martin Collinson, John D. West

**Affiliations:** a Centre for Integrative Physiology, University of Edinburgh Medical School, Hugh Robson Building, George Square, Edinburgh EH8 9XD, UK; b Medical and Developmental Genetics Section, MRC Human Genetics Unit, MRC IGMM, University of Edinburgh, Western General Hospital, Crewe Road, Edinburgh, EH4 2XU, UK; c School of Medical Sciences, Institute of Medical Sciences, University of Aberdeen, Foresterhill, Aberdeen, AB25 2ZD, UK

**Keywords:** Cornea, Limbal epithelial stem cells, Limbus, Lineage tracing, Stem cell quiescence, Stem cells

## Abstract

The limbal epithelial stem cell (LESC) hypothesis proposes that LESCs in the corneal limbus maintain the corneal epithelium both during normal homeostasis and wound repair. The alternative corneal epithelial stem cell (CESC) hypothesis proposes that LESCs are only involved in wound repair and CESCs in the corneal epithelium itself maintain the corneal epithelium during normal homeostasis. We used tamoxifen-inducible, CreER-*loxP* lineage tracing to distinguish between these hypotheses. Clones of labelled cells were induced in adult CAGG-CreER;R26R-*LacZ* reporter mice and their distributions analysed after different chase periods. Short-lived clones, derived from labelled transient amplifying cells, were shed during the chase period and long-lived clones, derived from stem cells, expanded. At 6 weeks, labelled clones appeared at the periphery, extended centripetally as radial stripes and a few reached the centre by 14 weeks. Stripe numbers depended on the age of tamoxifen treatment. Stripes varied in length, some were discontinuous, few reached the centre and almost half had one end at the limbus. Similar stripes extended across the cornea in CAGG-CreER;R26R-mT/mG reporter mice. The distributions of labelled clones are inconsistent with the CESC hypothesis and support the LESC hypothesis if LESCs cycle between phases of activity and quiescence, each lasting several weeks.

## Highlights

• We used lineage tracing to evaluate alternative corneal epithelial stem cell models.
• Clones emerged from the periphery and extended centripetally as radial stripes.
• This implies that active stem cells are at the periphery, not throughout the cornea.
• Labelling patterns suggested stem cells cycle through quiescent periods.
• More labelled clones were induced by treating younger mice.

## Graphical abstract

**Figure:**
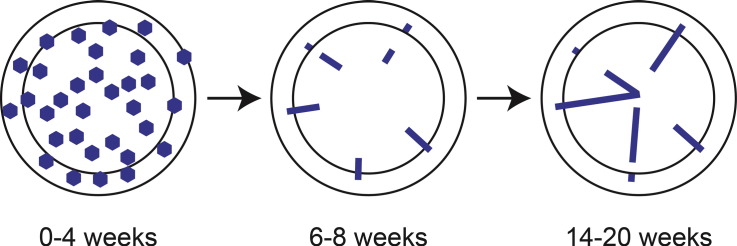

## 1 Introduction

Two hypotheses have been proposed to explain how stem cells maintain the corneal epithelium. The limbal epithelial stem cell (LESC) hypothesis proposes that all the stem cells are in the basal epithelial layer of the limbus, which lies between the cornea and conjunctiva (Schermer et al., 1986). The LESCs remain in the limbus, where they replace themselves and generate transient (or transit) amplifying cells (TACs) in the basal layer. The TACs divide, move into the cornea and then move centripetally across the corneal radius. They also produce more differentiated cells, which do not divide but leave the basal layer, move vertically through the suprabasal layers and are shed from the surface.

The alternative corneal epithelial stem cell (CESC) hypothesis (Majo et al., 2008) accepts that LESCs exist but proposes that these are only used to repair wounds and that, during normal homeostasis, the corneal epithelium is maintained by stem cells, which are present throughout the corneal epithelium. The original version of this hypothesis also proposed that, during normal homeostasis, cell movement was centrifugal rather than centripetal. Evidence for and against both hypotheses has been reviewed elsewhere (Sun et al., 2010, Mort et al., 2012, Yoon et al., 2014, West et al., 2015) and is summarised below.

### 1.1 Stem cells in the limbal epithelium

There is ample indirect evidence that stem cells are present in the limbal epithelium. ABCB5 is a promising new candidate LESC marker (Ksander et al., 2014) and older markers have been reviewed elsewhere (Mort et al., 2012). However, most markers are likely to identify early generation TACs as well as LESCs. In many tissues, slow-cycling stem cells are identifiable as label-retaining cells and, although this is not specific for stem cells, the presence of label-retaining cells among limbal, but not corneal, epithelial cells suggests that stem cells are in the limbus (Cotsarelis et al., 1989, Lehrer et al., 1998). Side population cells are characteristic of stem cells that express ABCG2 or other transporters, so the identification of some limbal epithelial cells as side population cells supports the existence of LESCs (Chen et al., 2004, Watanabe et al., 2004). Stem cells have a greater proliferative potential than TACs and in vitro colony-forming assays showed that human limbal, but not corneal, epithelium contains cells capable of producing large colonies (holoclones) (Pellegrini et al., 1999), again suggesting some limbal epithelial cells are stem cells.

There is strong experimental evidence from real-time studies in adult mice that, during normal homeostasis, corneal epithelial cells move centripetally, across the whole corneal radius, from the limbus or cornea periphery (Buck, 1985, Nagasaki and Zhao, 2003, Di Girolamo et al., 2015), as predicted by the LESC hypothesis. Evidence from mouse and rat mosaics and chimeras also shows that an initially randomly orientated mosaic patchwork switches to radial stripes in the early postnatal corneal epithelium (Collinson et al., 2002, Endo et al., 2007, Zhang et al., 2008, Mort et al., 2009, Hayashi et al., 2012, Iannaccone et al., 2012). The simplest interpretation is that, once LESCs are activated postnatally, clones of cells emerge from the limbus and extend centripetally, as predicted by the LESC hypothesis, to replace the original patchwork pattern.

Both the LESC and CESC hypotheses accept that LESCs exist but to distinguish between them we need to determine whether there are stem cells in the corneal epithelium itself and if LESCs play a role in corneal epithelial maintenance in the absence of wounding.

### 1.2 Do stem cells exist in the central corneal epithelium?

There is no definitive evidence for or against the existence of CESCs. For example, differences in marker expression, between the corneal and limbal basal epithelia, do not exclude the existence of CESCs that express different markers from LESCs. Likewise, the absence of label-retaining cells from the corneal epithelium does not rule out the existence of CESCs that are not slow cycling. Conversely, the identification of side population cells in the rat corneal epithelium is not good evidence that CESCs exist because, unlike those in the limbal epithelium, they do not express ABCG2 (Umemoto et al., 2005). Some corneal epithelial cells appear to have the high proliferative potential expected of stem cells. For example, the central corneal epithelia of rabbits (Huang and Tseng, 1991, Kawakita et al., 2011), mice (Majo et al., 2008) and humans (Dua et al., 2009) contain cells that can act as progenitors and maintain the tissue for some time if the limbus is eliminated, disconnected or lacks stem cells. Also, cultured pig corneal epithelial cells can produce holoclones (Majo et al., 2008) and human corneal epithelial cells can produce clonogenic spheres (Chang et al., 2011). However, it remains unclear whether these cells act as progenitors during normal homeostasis in vivo (as the CESC hypothesis predicts) or if their proliferative potential is normally latent but can be unmasked in culture or when LESCs are unable to maintain the corneal epithelium in vivo.

### 1.3 Do LESCs maintain the corneal epithelium during normal homeostasis?

Compelling evidence for centripetal movement argues against the original CESC hypothesis, because it proposed centrifugal cell movement, but it would not exclude a modified CESC hypothesis with centripetal movement. Other evidence about whether LESCs maintain the corneal epithelium during normal homeostasis is conflicting. The postnatal switch, from a random mosaic pattern to radial stripes, described above, supports the LESC hypothesis. In contrast, when labelled limbal tissue was transplanted surgically to an unlabelled mouse limbus, the transplant failed to colonise the corneal epithelium unless the host corneal epithelium was removed (Majo et al., 2008). This result is inconsistent with the LESC hypothesis and prompted the authors to propose the CESC hypothesis. As these two experiments lead to opposite conclusions about whether LESCs replenish the corneal epithelium during normal homeostasis, more definitive evidence is needed.

### 1.4 Aims

The primary aim of this project was to directly test the LESC and CESC hypotheses and, in particular, determine whether any cells in the corneal epithelium can generate long-lived clones during normal homeostasis. We used tamoxifen-inducible CreER-*loxP* lineage tracing with adult CAGG-CreER;R26R-*LacZ* reporter mice, without surgical intervention. The ubiquitous CAGG promoter provided unbiased, stochastic labelling of all cell types, including putative stem cells in the limbus or cornea. The observed distribution of long-lived clones, founded by labelled stem cells, was inconsistent with the CESC hypothesis, but supports the LESC hypothesis if LESCs cycle through phases of activity and quiescence.

## 2 Materials and methods

### 2.1 Mice

Animal work was approved by the University of Edinburgh Ethical Review Committee and performed in accordance with UK Home Office regulations under project license PPL60/4302. CAGG-CreER;R26R-*LacZ* mice were bred by crossing homozygous R26R-*LacZ* (B6.129S4-*Gt*(*ROSA*)*26Sor*^*tm1Sor*^/J) Cre-reporter mice (Soriano, 1999) on a C57BL/6J genetic background to hemizygous CAGG-CreER mice (Tg(CAG-cre/Esr1*)5Amc) (Hayashi and McMahon, 2002) on a predominantly CBA/Ca background (N7). The *LacZ* reporter transgene expresses β-galactosidase (β-gal) in the presence of nuclear-located Cre, so expression is induced when tamoxifen binds to the modified oestrogen receptor, ER, and moves CreER to the nucleus. CAGG-CreER;R26R-mT/mG mice were bred by crossing homozygous R26R-mT/mG (Gt(ROSA)26Sor^tm4(ACTBtdTomato,-EGFP)Luo^) Cre-reporter mice (Muzumdar et al., 2007) to hemizygous CAGG-CreER mice and both were on a C57BL/6 genetic background. In the presence of nuclear-located Cre, the mT/mG reporter switches fluorescent colours from red (membrane-targeted tandem dimer Tomato; mT) to green (membrane-targeted green fluorescent protein; mG). Mice were genotyped for Cre by PCR using forward primer 5′-TCGCAAGAACCTGATGGAC-3′ and reverse primer 5′-CCCAGAAATGCCAGATTAC-3′.

### 2.2 Tamoxifen treatment

Tamoxifen (Sigma-Aldrich) was freshly dissolved in corn oil (20–35 mg/ml) with sonication in a 40 °C water bath, prior to injection. Mice were weighed and tamoxifen doses (100 μg/g body weight) were calculated for each mouse. CAGG-CreER;R26R-*LacZ* mice, aged 4, 12 or 24 weeks, were injected with tamoxifen on three consecutive days. Most CAGG-CreER;R26R-mT/mG mice were injected with tamoxifen at 12 weeks on three consecutive days but, for a pilot study, some were injected on five consecutive days. Negative control mice were not injected. After the appropriate chase period, mice were culled by cervical dislocation, following overdose of gaseous halothane anaesthetic, and eyes were enucleated.

### 2.3 β-Galactosidase staining and measurement of corneal stripes

Eyes were fixed in 0.2% glutaraldehyde for 2 h, stained for β-gal with X-gal, post-fixed in 4% paraformaldehyde (PFA) and stored in 70% ethanol at 4 °C as previously described (Collinson et al., 2002). This whole-mount method stains the ocular epithelia but the corneal stroma remains unstained unless the epithelium is damaged. Calibrated digital images were captured with an Axiovision 4.8 digital camera system on a Wild M5A dissecting microscope. The length of each β-gal positive stripe was measured in calibrated photographs of stained eyes, with Adobe Photoshop software, as previously described (Douvaras et al., 2012). Multiple stripes that were radially aligned across the cornea were counted as one discontinuous stripe. For corneal stripes, which did not reach the limbus (cornea–cornea or CC stripes), the radial distance from the limbus to the peripheral end of the stripe was also measured. For discontinuous stripes, each β-gal positive and β-gal negative region was measured separately. Subsequently, some eyes were embedded in wax, sectioned at 7 μm and counterstained with eosin.

### 2.4 Confocal imaging of flattened whole-mount corneas

Eyes were fixed in 4% PFA overnight and transferred to phosphate buffered saline. Corneas were excised under a dissecting microscope, flattened by making 2–4 radial cuts, mounted in Vectashield (epithelium up) on a glass slide and sealed under a coverslip. They were imaged on a Zeiss LSM 510 confocal inverted microscope using a dry 20 × and oil immersion 40 × and 63 × objectives. Tiled montages, with no overlap between frames, and confocal Z-stacks were acquired.

### 2.5 Statistical analysis

GraphPad Prism 5 was used for most statistical tests, as described in the text. An online statistical calculator (http://vassarstats.net/index.html) was used for chi square goodness of fit tests and 3 × 2 Fisher's exact tests.

## 3 Results

### 3.1 Lineage tracing of β-gal labelled clones induced at 12 weeks

**Fig. 1:**
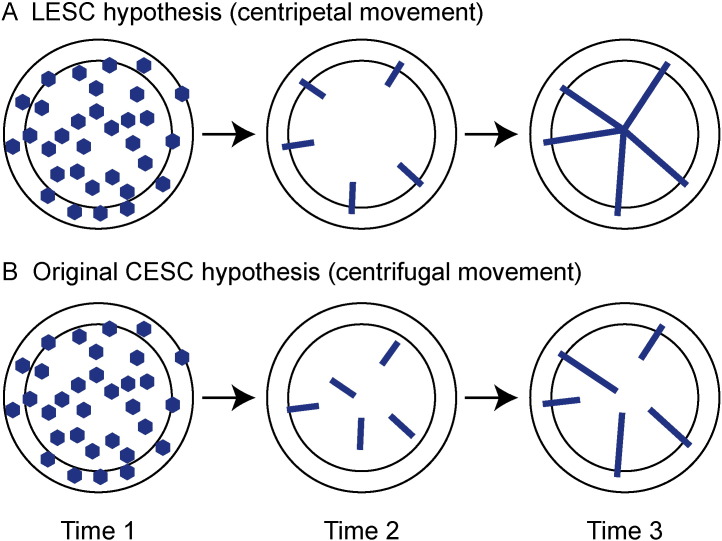
Original predictions for lineage tracing with a tamoxifen-inducible, ubiquitous reporter. Inner discs and outer rings represent the corneal and limbal epithelia respectively; blue areas represent labelled clones.

As labelling is unbiased and stochastic, initially a proportion of all cell types will be β-gal positive and both limbal and corneal epithelial stem cells (if they exist) should be labelled in the reporter mice. Patterns of labelling predicted by the LESC and CESC hypotheses are shown in Fig. 1A and B. Short-lived labelled clones, produced by labelled TACs, should be shed from the corneal epithelium, leaving long-lived labelled clones, produced by labelled stem cells. The LESC hypothesis predicted that that each long-lived clone would have one end in the limbus and extend centripetally to the centre of the corneal epithelium to form a radial stripe. The original CESC hypothesis predicted that long-lived clones would extend centrifugally from stem cells, located throughout the corneal epithelium itself. Such clones may not span the entire radius.

**Fig. 2:**
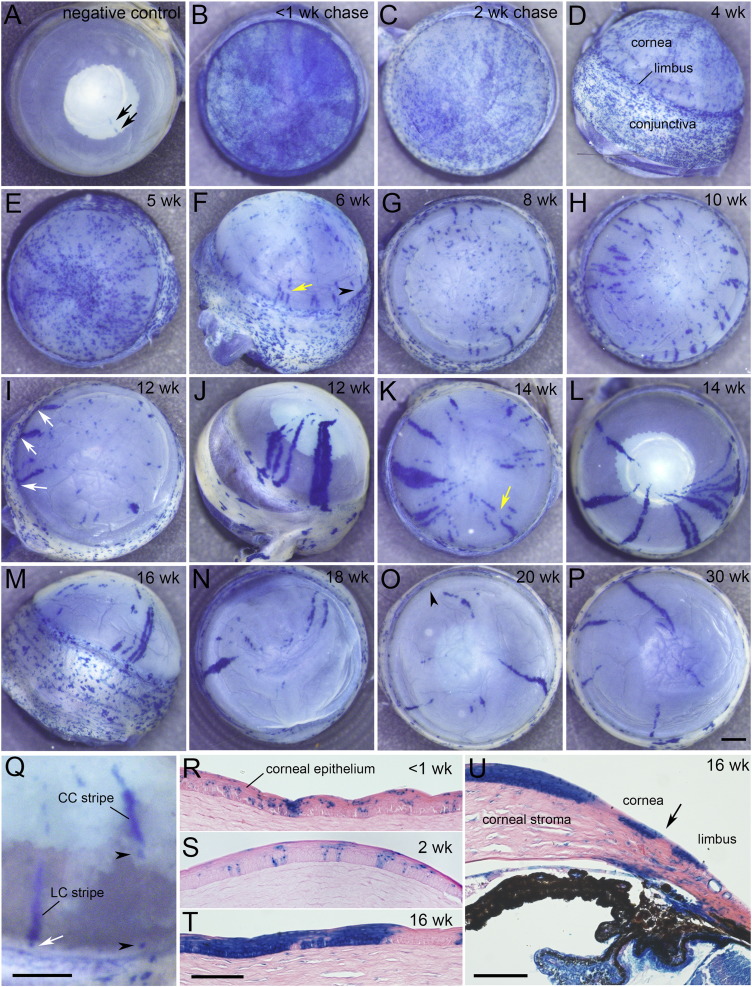
Lineage tracing with β-gal labelled clones induced at 12 weeks in CAGG-CreER;ROSA26R-*LacZ* mice. (A) One of 14 eyes from uninjected negative controls showed blue β-gal staining (arrows). (B–P) Eyes from mice, injected with tamoxifen at 12 weeks and stained for β-gal activity after various chase periods. (B) 5-Day chase showing initial diffuse blue β-gal staining. (C–E) After 2–5 weeks (wk), there were small, β-gal positive patches throughout the cornea and conjunctiva. (F–P) From 6 weeks, stripes extended radially across the cornea. (Q) Limbal–corneal (LC) and corneal–corneal (CC) stripes. White arrows in I & Q show limbal ends of LC stripes. Black arrowheads in F, O & Q show gaps between labelled limbal regions and radially aligned CC stripes in the cornea. Yellow arrows in F & K show gaps between two radially aligned stripe regions (discontinuous stripes). (R–U) Histological sections of β-gal-stained corneas after chase periods of 5-days (R), 2 weeks (S) and 16-weeks (T, U). Arrow in U shows approximate limbal–corneal boundary. Scale bars: P (for A–P) & Q, 0.5 mm; T (for R–T) & U, 100 μm.

In the main lineage tracing study, CAGG-CreER;R26R-*LacZ* mice were used to characterise the β-galactosidase (β-gal) histochemical staining pattern in the cornea. Mice were injected with tamoxifen three times at 12 weeks, to induce *LacZ* expression, and left for chase periods of up to 20 weeks before staining for β-gal activity (8–14 corneas from 4–7 mice per group). Another four corneas (from three mice) were analysed after a 30-week chase period to test whether labelled clones were longer-lived, but this small group was not included in the quantitative analysis. The results are illustrated in Fig. 2.

All six eyes from 12-week old uninjected negative control CAGG-CreER;R26R-*LacZ* mice and 7/8 eyes from 24-week old negative controls showed no β-gal corneal staining. However, one eye had two small, radially aligned, β-gal positive stripes (Fig. 2A). This suggests that leaky *LacZ* reporter expression occurred in some negative control eyes (presumably caused by unexpected Cre nuclear localisation (Haldar et al., 2009)). However, the staining frequency (1/14; 7%) was much lower than after tamoxifen injection (147/158 eyes; 93%). Leaky reporter expression in adult stem cells before tamoxifen-treatment would induce some long-lived labelled clones earlier than expected. However, most leaky expression would be in TACs or more differentiated cells, which would not produce long-lived clones. Leaky expression, in stem cell progenitors, during development would label groups of clonally related stem cells so produce multiple stripes, possibly in both eyes.

All 12 corneas, which were stained less than one week after tamoxifen injection, had diffuse blue staining over the ocular surface (Fig. 2B) and histology showed that most β-gal positive corneal epithelial cells were distributed sporadically but rarely formed clones that spanned the whole epithelial thickness (Fig. 2R). This is consistent with the expectations that reporter transgene expression would be induced stochastically in differentiated cells, TACs and stem cells. After chase times of 2–5 weeks, the diffuse staining was more discrete and numerous small β-gal positive patches were present (Fig. 2C–E) and many spanned the full epithelial thickness (Fig. 2S). At 0–5 weeks the intensity of β-gal positive staining sometimes varied in different corneal sectors but this differed from the distinct radial stripes that formed after longer chase times. The frequency of small patches gradually declined after longer chase periods, consistent with the loss of short-lived clones from labelled TACs. At 6 weeks, short radial stripes appeared at the periphery of the cornea (Fig. 2F). Longer radial stripes were present, after chase periods of 8 to 30 weeks (Fig. 2G–P) and their survival until at least 30 weeks implies they were long-lived clones. Histology showed that most β-gal positive corneal epithelial stripes spanned the full epithelial thickness (Fig. 2T, U).

Initially, the conjunctiva was covered in β-gal positive spots, which enlarged to form circumferentially elongated patches (Fig. 2D, F, M). This is consistent with evidence, from other mosaic systems, that the conjunctiva is maintained by its own stem cells (Collinson et al., 2002, Mort et al., 2009, Nagasaki and Zhao, 2005).

**Fig. 3:**
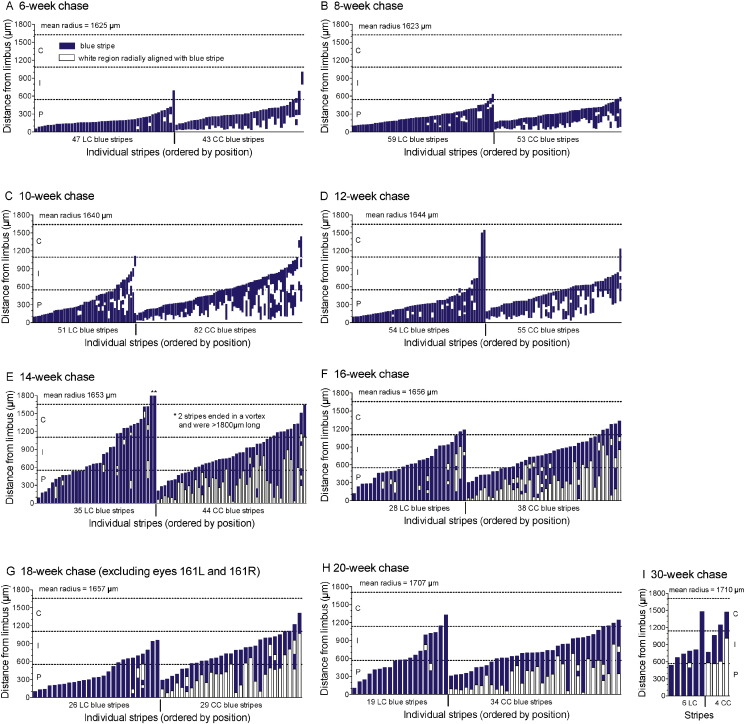
Lengths and radial positions of individual β-gal positive stripes induced at 12 weeks. (A–I) The positions and lengths of β-gal positive stripes and radially aligned unstained areas are shown relative to the limbus. The top horizontal line in each chart indicates the mean radius for the group and the other two lines divide the radius into equal lengths to define peripheral (P), intermediate (I) and central (C) regions. LC stripes (with one end at the limbus) are shown separately from CC stripes (which do not include the limbus) and stripes are ordered by position. Eyes 161L and 161R were excluded from the analysis; see text and Fig. S1.

**Fig. 4:**
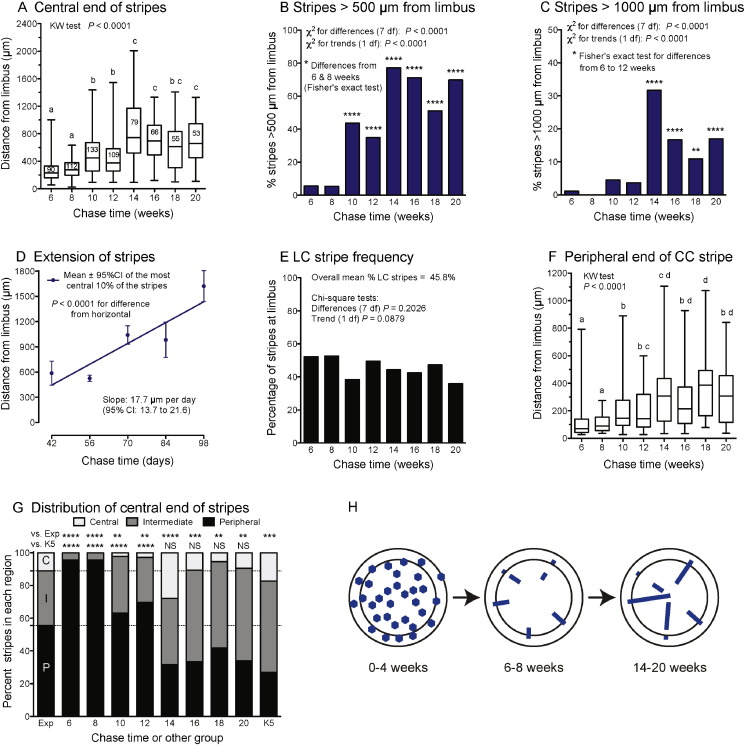
Effects of chase time on β-gal positive stripes induced at 12 weeks. (A) Comparisons of the distance between the limbus and the central end of β-gal-positive stripes by Kruskal–Wallis (KW) test and Dunn's multiple comparison tests. (Shared letters indicate no significant difference; for other comparisons, *P* < 0.01.) (B, C) Percentage of stripes with their central end > 500 μm (B) or > 1000 μm (C) from the limbus after different chase times. (D) Distance between the limbus and the central end of the most central 10% of the β-gal stripes at different chase times. Linear regression showed stripes extended centripetally between 6 and 14 weeks. (E) The percentage of stripes that were LC stripes at different chase times. (F) Comparisons of the distance between the limbus and the peripheral end of CC stripes by Kruskal–Wallis and Dunn's tests. (For comparisons without shared letters, *P* < 0.05.) (G) Comparison of the percentage of the central ends of β-gal stripes, in the peripheral (P), intermediate (I) and central (C) regions of the cornea, after different chase times (see text for explanation). The “Exp” bar shows the expected P:I:C frequency distribution if the central ends were randomly distributed over the frontal view of the cornea. The “K5” bar shows the P:I:C distribution previously reported for adult mosaic, *KRT5*^*LacZ*/−^ transgenic mice (Douvaras et al., 2012). For each chase time (6–20 weeks) and “K5”, the observed P:I:C distributions differed significantly from the expected proportions (“vs. Exp”) by goodness of fit chi-square test. From 14–20 weeks, the P:I:C distributions did not differ significantly from “K5” (“vs. K5”) by Fisher's exact test. (H) Interpretation of observed β-gal labelling patterns. In the box and whisker plots in A, F and other figures, the whiskers show the minimum and maximum values. The numbers of stripes analysed in A–G, are shown within the boxes in A. CI, confidence interval; NS, not significant; ** *P* < 0.01; *** *P* < 0.001; **** *P* < 0.0001.

Five eyes from three mice had multiple long stripes (Fig. S1). After an 18-week chase, eyes 161L and 161R from the same mouse had numerous wide stripes, similar to those in chimeras and X-inactivation mosaics (Collinson et al., 2002, Mort et al., 2009), and the conjunctival patches were also unusually large (Fig. S1E-G). This strongly suggested that leaky reporter expression had occurred in stem cell precursors during development and that wide stripes were produced by clusters of clonally related stem cells. The other three eyes (49R, 39L and 39R; Fig. S1B-D) had long stripes and larger conjunctival patches than most (but not all) others at similar chase times (Figs. 2M and S1H). Eyes 161L and 161R were excluded from the quantitative analysis but, as it was uncertain whether any stripes in the other three eyes were caused by leaky transgene expression, the quantitative analysis was repeated with these eyes (Fig. 3, Fig. 4 and S2) and without them (Supplementary Fig. S1, Supplementary Fig. S2, Supplementary Fig. S3). The stripe number per cornea was positively correlated between left and right eyes but there were no major differences in stripe numbers among different chase periods, either with or without eyes 49R, 39L and 39R (Fig. S2). Stripe lengths varied widely at all chase times (Fig. 3).

Superficially consistent with both LESC and CESC hypotheses, some stripes had one end at the limbus (limbus–cornea or LC stripes; white arrows in Fig. 2I, Q) whereas others had both ends in the cornea (cornea–cornea or CC stripes). Overall, 46% of stripes were LC stripes. CC stripes were often aligned with a stained region in the limbus (arrowheads in Fig. 2F, O, Q), suggesting they may be produced by labelled LESCs. Sometimes, two or more stripes were radially aligned (yellow arrows in Fig. 2F, K). As there were only a few stripes per cornea, it is unlikely that radially aligned stripes arose independently so they were classified as single, discontinuous stripes. Discontinuous stripes occurred among both LC and CC stripes. Some long stripes changed direction abruptly near the centre to form a central whorl (Fig. S1B, D, F), as seen in some chimeras and X-inactivation mosaics (Collinson et al., 2002, Mort et al., 2009), suggesting that guidance cues directing cell movement change near the centre of the cornea.

### 3.2 Direction of stripe extension and location of stem cells

To determine whether the clonal patterns were most consistent with an LESC or CESC explanation for corneal maintenance, a quantitative analysis was performed. The changing position of the central end of the stripes showed that stripes extended from the periphery towards the centre during chase times of 6 to 14 weeks and then remained fairly stable (Fig. 4A). This was confirmed by comparisons of the percentage of stripes with central ends greater than 500 μm or 1000 μm from the limbus (Fig. 4B, C). Fig. 4D shows a crude estimate of the mean rate of centripetal stripe extension during the 6–14 week interval, based on the mean position of the 10% of stripes that were closest to the centre of the cornea, but this will be imprecise because stripe lengths were very variable at all time points. In contrast to these results, the original CESC hypothesis predicted that stripes should extend towards the limbus so some CC stripes would reach the boundary with the limbus and become LC stripes. However, the proportion of LC stripes did not increase (Fig. 4E) and the distance between the limbus and the more peripheral end of the CC stripes did not decrease with chase time (Fig. 4F).

The CESC hypothesis also proposed that active stem cells were distributed throughout the corneal epithelium. The peripheral (P), intermediate (I) and central (C) regions, shown in Fig. 3, form concentric areas in the intact eye. These were defined by dividing the frontal view of the corneal radius into three equal lengths so their relative areas were 55.56% P, 33.33% I and 11.11% C (Douvaras et al., 2012). Although part of the peripheral cornea is foreshortened in the frontal view of the cornea, the relative areas still apply to the visible portion. If clones extended centrifugally from stem cells, distributed randomly over the frontal view of the cornea, the central ends of stripes would be expected to be distributed according to the P:I:C area ratio shown in the first bar (“Exp”) in Fig. 4G. For 6 to 20 week chase times, the observed frequencies of the central stripe ends in P, I and C regions differed significantly from the expected proportions (Fig. 4G), confirming that stripes did not extend centrifugally from randomly distributed stem cells. Differences in P:I:C central stripe positions among the different chase times are consistent with centripetal extension of stripes from the periphery, between 6 and 14 weeks, as shown in Fig. 4A–F.

The published P:I:C distribution for β-gal positive stripes in adult, mosaic, *KRT5*^*LacZ*/−^ transgenic mice (Douvaras et al., 2012) is shown in the last bar (“K5”) of Fig. 4G. For 14–20 week chase times, the observed P:I:C frequency distributions did not differ significantly from those of adult *KRT5*^*LacZ*/−^ transgenic mice, even though *KRT5*^*LacZ*/−^ mosaicism is thought to arise much earlier (by stochastic transgene silencing early in development) than our tamoxifen-induced labelling. This suggests that the P:I:C frequency distributions for 14–20 week chase times represent the mature pattern of radial stripe positions. Thus, contrary to the predictions for Time 3 in Fig. 1A, the mature stripe pattern in CAGG-CreER;R26R-*LacZ* corneas includes CC stripes and discontinuous stripes.

The labelling patterns shown in Fig. 2, Fig. 3, Fig. 4 are summarised in Fig. 4H and differ from both initial predictions (Fig. 1A, B). Similar results to those in Fig. 4A–G were obtained in a parallel analysis, which excluded the three corneas with possible leaky reporter expression (Fig. S3).

### 3.3 Stem cell numbers

The number of active stem cells that maintain the corneal epithelium can be estimated as the number of stripes, induced in adults, which would fit around the limbal circumference if they were evenly distributed. For stripes induced at 12 weeks, the ratio of the modal stripe width at the limbal–corneal boundary (35–40 μm) and the overall mean circumference (10,365 μm) implies that 259–296 stripes would fit around the limbal circumference (Fig. S4A, B). A similar circumference/stripe width ratio was obtained using separate circumference measurements for each eye (Fig. S4C, D). There were minor differences in stripe widths among different chase times (or ages) but no consistent trend for stripe widths to increase with chase time (or age) (Fig. S4E, F). Similar estimates of the number of stripes that would fit around the limbal circumference were obtained from stripes induced at 4 weeks (Fig. S5).

### 3.4 Lineage tracing of clones labelled with a fluorescent marker

**Fig. 5:**
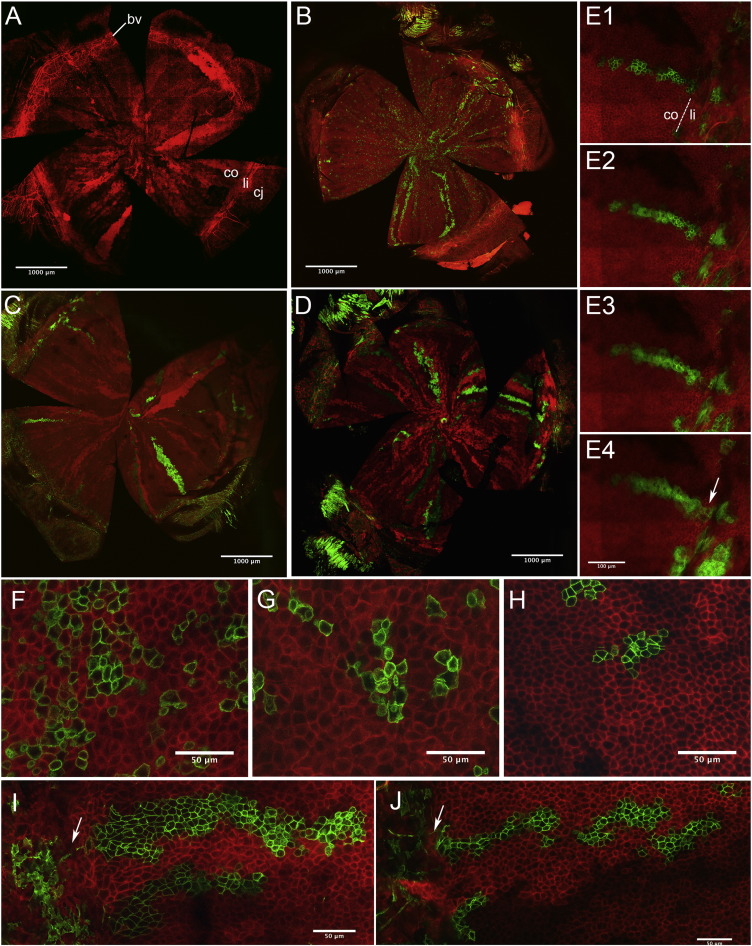
Confocal images of GFP-positive clones induced at 12 weeks in CAGG-CreER;R26R-mT/mG mice (A) Flat-mounted cornea (co), limbus (li) and conjunctiva (cj) from a 12-week old uninjected negative control mouse showing no GFP-positive clones (bv, limbal blood vessels). (B–J) GFP-positive patches and stripes in the corneal epithelium after 3 (B, C, E–J) or 5 (D) tamoxifen injections. (B) 4-Day chase, showing small GFP-positive patches, induced by tamoxifen, and GFP-positive radial stripes, implying leaky expression. (C, D) Radial GFP-positive stripes after 35-week (C) and 21-week (D) chase periods. (E1–4) Images from a confocal Z-stack (8-week chase) showing a GFP-positive stripe in the peripheral cornea connected to GFP-positive cells in the limbus in E4 (arrow). (F-H) GFP-positive patches in the corneal epithelium after 5-day (F), 4-week (G) and 8-week (H) chase periods. (I, J) GFP-positive stripes in the peripheral corneal epithelium (arrows indicate the limbus). Scale bars: 1000 μm (A–D); 100 μm (E); 50 μm (F–J).

Corneas, from tamoxifen-injected CAGG-CreER;R26R-mT/mG mice, were analysed by confocal microscopy after various chase times. Uninjected negative controls, analysed at 12 and 24 weeks (6 corneas from 3 mice at each age), showed no GFP-positive fluorescence (mG) but the level of red fluorescence (mT) varied, indicating variable expression of the mT/mG reporter transgene (Fig. 5A). However, radial GFP-positive stripes were observed in 8 of 13 corneas, less than one week after 3 tamoxifen injections (Fig. 5B), implying that the mT to mG switch occurred spontaneously at a significant frequency. Thus, for longer chase periods, we could not determine whether labelling was initiated at or before tamoxifen injection, so we only used the fluorescent images to illustrate the pattern of labelling. Small GFP-positive patches in the corneal epithelium declined in frequency from 2 to 8 weeks after labelling (Fig. 5F–H). Many corneas had radial striped patterns, similar to the β-gal positive stripes in the main study and CC, LC and discontinuous stripes were all seen. Radial stripes were present after chase times of up to 35 weeks (Fig. 5C), and were also produced in a pilot study with a higher dose of tamoxifen (Fig. 5D). Moreover, 3-dimensional confocal analysis of some corneal stripes that reached the limbal–corneal boundary showed that they were connected to the limbus in the basal layer even if this was not always apparent in the suprabasal layers (Fig. 5E). This confirms that LC stripes extend into the limbus and are not restricted to the peripheral cornea.

Stripes close to the limbus were typically 2–5 cells wide (Fig. 5I, J). Together with the published estimate of 424 basal cells across the corneal diameter in 12-week old mice (Douvaras et al., 2013), this provides an alternative, crude estimate that about 207–519 stripes would fit around the corneal circumference. (The measured corneal “diameter” was an arc and the arc/chord ratio of the mouse cornea (Fig. 19.1a in Mort et al., 2012) implies that the equivalent chord length would be approximately 330 cells, which predicts there are about 1037 cells around the limbal–corneal circumference.)

### 3.5 Effects of inducing β-gal labelled clones at different ages

**Fig. 6:**
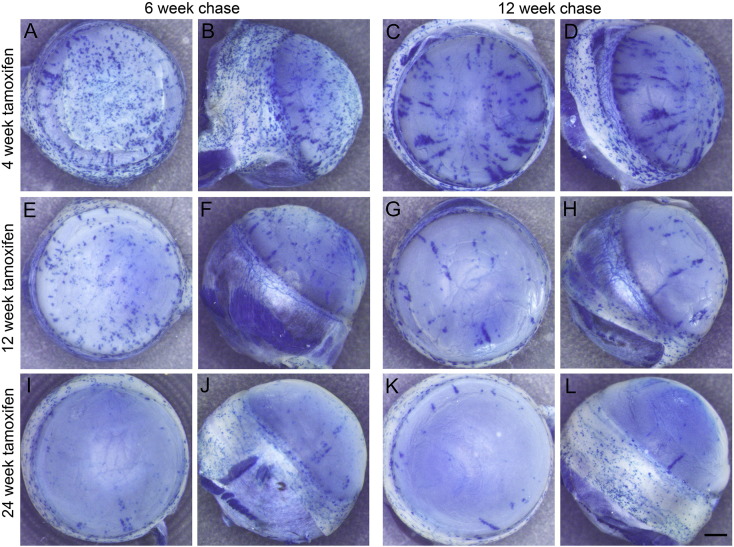
Lineage tracing of β-gal labelled clones induced at different ages. β-gal-stained corneas from mice that were injected with tamoxifen 3 times at 4 (A–D), 12 (E–H) or 24 (I–L) weeks of age and chased for 6 or 12 weeks. Front and side views are shown of the same eyes. (Eyes E–H are part of the series illustrated in Fig. 2.) Scale bar, 0.5 mm.

**Fig. 7:**
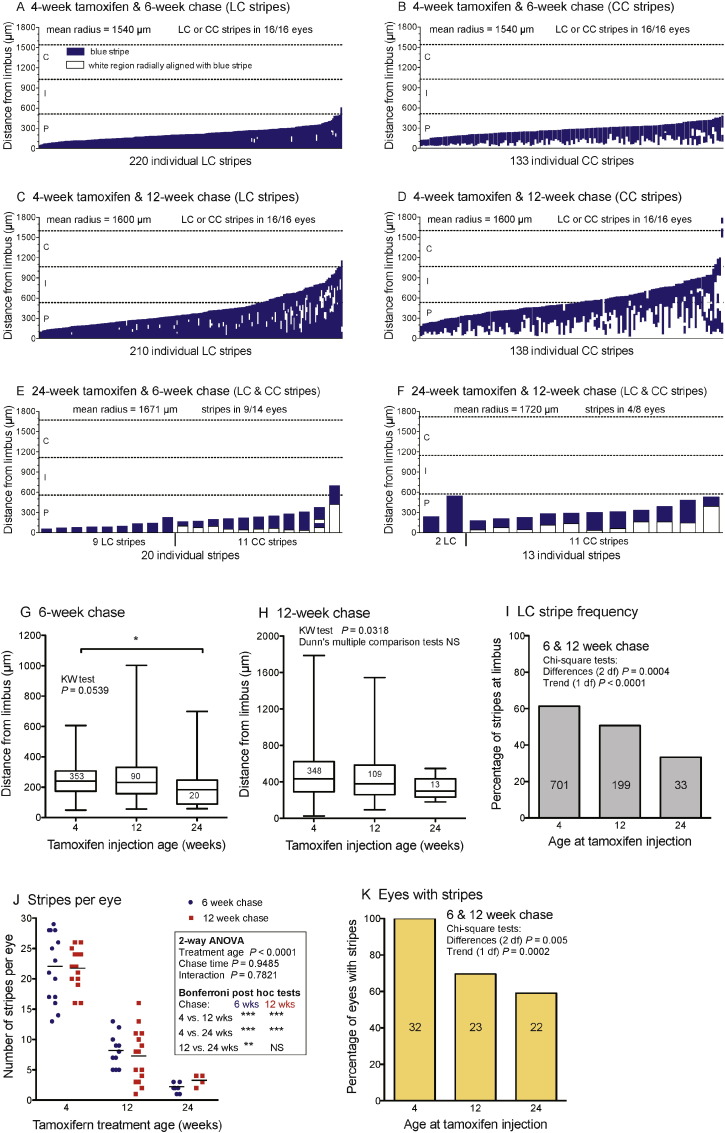
Quantitative comparisons of β-gal positive stripes induced at different ages. (A–F) Positions and lengths of β-gal positive stripes and radially aligned unstained areas, in corneas from mice treated with tamoxifen at 4 (A–D) or 24 (E, F) weeks and chased for 6 or 12 weeks. (G, H) Distance between the limbus and the central end of β-gal stripes induced by 3 tamoxifen injections at 4, 12 or 24 weeks for 6-week (G) and 12 week (H) chase periods by Kruskal–Wallis (KW) test and Dunn's multiple comparison test. (I) The percentage of stripes that were LC stripes for mice injected with tamoxifen at different ages. (J) Comparisons of the numbers of stripes per eye for mice injected at different ages. The results of a 2-way analysis of variance (ANOVA) and Bonferroni post-hoc tests are shown in the figure. (K) The percentage of eyes with stripes for mice injected with tamoxifen at different ages. In G–K, the data for mice treated at 12 weeks were from the series shown in Fig. 2, Fig. 3, Fig. 4 and included eye 49R. The numbers in the bars or boxes are stripe numbers (G, H & I) or eye numbers (K). NS, not significant; * *P* < 0.05; ** *P* < 0.01; *** *P* < 0.001.

Some CAGG-CreER;R26R-*LacZ* mice were injected with tamoxifen 3 times at either 4 or 24 weeks and left for chase periods of 6 or 12 weeks. Stripe patterns were compared to those injected at 12 weeks in the main series (Fig. 6). The age of tamoxifen treatment had no major effect on the position of the central end of stripes after chase periods of 6 or 12 weeks (Fig. 7A–H) so there was no evidence that age affected the rate of stripe extension. However, there were significant trends for younger-treated mice to have more LC stripes (Fig. 7I), more stripes overall (Fig. 7J) and fewer eyes without stripes (Fig. 7K), even though the dose was corrected for body weight. There also appeared to be more small patches in younger mice after a 6-week chase (Fig. 6) but this was not quantified. The stripe number per cornea was positively correlated between left and right eyes for mice treated at 4 weeks but not for the smaller group treated at 24 weeks (Fig. S5B).

## 4 Discussion

### 4.1 Comparisons with other cornea CreER-*loxP* lineage tracing experiments

Our main aim was to use inducible CreER-*loxP* lineage tracing, without disturbing homeostasis, to test whether long-lived clones of corneal epithelial cells arose in the adult mouse limbus or central cornea and so distinguish between the LESC and CESC hypotheses. While our study was in progress, two other corneal epithelial CreER-*loxP* lineage-tracing studies were reported (Di Girolamo et al., 2015, Amitai-Lange et al., 2015). Both these investigations used tamoxifen-inducible K14-CreER^T2^;R26R-confetti transgenic mice. As K14-CreER^T2^ was used to drive reporter expression, they did not label all cell types and could not determine whether CESCs exist and contribute to corneal epithelial maintenance. Keratin 14 (K14) is expressed in the basal limbal epithelium but the two studies reported different extents of K14 expression in the corneal epithelium. Thus, our study is the first to demonstrate that CESCs either do not exist or do not contribute significantly to corneal epithelial maintenance under normal homeostatic conditions.

In an elegant real-time experiment, Di Girolamo et al. followed the same clones and demonstrated convincingly that fluorescent stripes emerged from the limbus by about 5 weeks and extended centripetally to the centre of the cornea by 17–21 weeks (Di Girolamo et al., 2015). The other study, by Amitai-Lange et al. used different mice for each chase time and reported that stripes emerged at approximately 1 month and extended to the centre by 4–5 months (Amitai-Lange et al., 2015), consistent with the real-time study. Our analysis also demonstrated that long-lived labelled clones emerged at the periphery and, in some cases, we showed that the ends of LC stripes at the limbal–corneal boundary were within the limbus.

All three investigations showed that the corneal epithelium is maintained by LESCs but our study extends this in three ways. First, unlike K14-CreER ^T2^, CAGG-CreER lineage tracing labels all cell types and we found no evidence that any long-lived clones emerged in the central cornea. This allowed us to exclude the possibility that the corneal epithelium is normally maintained by CESCs in the central cornea. Second, our analysis revealed that many stripes were disconnected from the limbus and/or discontinuous and this prompted a new prediction about LESC quiescence (discussed below). Third, we showed that younger mice produced more stripes in response to tamoxifen, which raises further questions about LESC behaviour.

Minor discrepancies among the three studies may be explained by technical differences. For example, in the other studies there was a shorter lag before stripes emerged (5 weeks vs. 6 weeks in our experiments) but stripes took longer to extend fully (17–21 weeks vs. 14 weeks). The initial lag could be due to delayed production of TACs from LESCs that are labelled during quiescence, retention of some limbal TACs to maintain the limbal epithelium and time required for limbal TACs to reach the cornea and produce an identifiable stripe. Our CAGG-CreER system produced more short-lived, labelled TAC clones, which may have hindered identification of the earliest stripes. In the other studies, mice were injected with tamoxifen at 6–8 weeks. To ensure corneal epithelial homeostasis had stabilised, we labelled most mice at 12 weeks but also compared labelling at 4 and 24 weeks. We found no significant effect of age on the rate of stripe extension so it seems unlikely that the use of younger mice contributed to the slower stripe extension in the other studies. This may simply reflect the difficulty in evaluating when stripe patterns become stabilised because, at least in our study, stripe lengths varied and few stripes reached the centre, even after long chase times.

Although CC stripes and discontinuous stripes were not specifically discussed in the other reports, examples of these and multi-coloured stripes were illustrated (e.g. Fig. 5 in Di Girolamo et al., 2015 and Figs. 3B and S2 in Amitai-Lange et al., 2015). With the confetti reporter system, multi-coloured stripes might arise in an equivalent way to discontinuous or CC stripes.

Amitai-Lange et al. calculated that about 90 stripes would fit into the cornea (Amitai-Lange et al., 2015), whereas we estimated that about 250–300 stripes could fit around the corneal circumference at the periphery. This provides a crude estimate of the number of active stem cells but the total stem cell number would be higher if some are quiescent. Our estimate also predicts there are 2–3 stem cells per coherent clonal group in X-inactivation mosaics, although these coherent clone numbers were more affected by age than our circumference/stripe width ratios (Collinson et al., 2002, Mort et al., 2009).

### 4.2 Discriminating among different stem cell hypotheses

The β-gal labelling patterns observed after different chase times in the main lineage tracing experiment (summarised in Fig. 4H) were more complex than expected and did not match our original predictions for the either hypothesis exactly (Fig. 1A, B). Although long-lived clones emerged at the periphery, as expected for the LESC hypothesis, many stripes were CC stripes and disconnected from the limbus. In principle, LESCs could produce CC stripes if some labelled LESCs are (i) lost or replaced by unlabelled neighbouring LESCs by stochastic neutral drift (Mort et al., 2012, Klein and Simons, 2011) or (ii) become quiescent. In many cases the peripheral end of a CC stripe was radially aligned with a labelled spot in the limbus, which argues in favour of quiescence rather than LESC loss or replacement, but a combination of quiescence and loss/replacement is also possible. For simplicity, we compared results of the lineage tracing study to expectations for four hypotheses: the original CESC hypothesis with centrifugal movement (CESC-cf), a modified CESC hypothesis with centripetal movement (CESC-cp), the LESC hypothesis with continuously slow cycling stem cells (LESC-cc) and a version of the LESC hypothesis with intermittent periods of quiescence (LESC-iq). The evidence is summarised in Table 1 and discussed below.

Most labelled clones were short lived, as predicted by all four hypotheses. Stripes emerged at the periphery and, at 6 and 8 weeks, they were almost all confined to the periphery (with few in the intermediate region and none in the centre). This is consistent with both versions of the LESC hypothesis but is strong evidence against both versions of the CESC hypothesis. Stripes extended towards the centre, which is strong evidence against the original CESC-cf hypothesis.

For Time 2 in Fig. 1A, B, we predicted that, soon after stripes emerged, either all stripes would be LC stripes (LESC-cc hypothesis) or almost all would be CC stripes (CESC-cf hypothesis). Contrary to both predictions, at 6–8 weeks about 50% of stripes were LC stripes. Radial alignment of CC stripes with β-gal positive spots in the limbus suggests they could be produced from a labelled LESC that had subsequently become quiescent (LESC-iq hypothesis). The presence of significant numbers of CC and LC β-gal positive stripes, soon after stripes formed, is consistent with the LESC-iq hypothesis but not LESC-cc. It might also be consistent with CESC-cf (if some CESCs were close enough to the limbus for stripes to reach the limbus by 6–8 weeks) and CESC-cp (if some CESCs were at the limbal–corneal boundary).

The distance between the limbus and the more peripheral end of the CC stripes did not decrease with longer chase times and the proportion of LC stripes did not increase. This is not consistent with CESC-cf (which predicts stripes should extend towards the limbus and convert some CC stripes to LC stripes) or LESC-cc (which predicts CC stripes should not exist) but is consistent with CESC-cp and LESC-iq.

We originally expected that most stripes would extend across the full radius if LESC-cc was correct but not if CESC-cf was correct (Fig. 1A, B). However, this was too simplistic because, if movement is centripetal, geometry dictates that stripes will narrow, as the corneal circumference shrinks, and many of those produced by single LESCs will terminate before reaching the centre. Gaps in stripes could be caused by stem cell quiescence (LESC-iq hypothesis) or incursion of neighbouring unlabelled cells. The presence of stripes that fail to reach the centre and discontinuous stripes are both consistent with all four hypotheses.

The distance between the central end of stripes and the limbus varied widely at all chase times. This is consistent with CESC-cf and CESC-cp if CESCs were scattered throughout the cornea. It is also consistent with LESC-iq because individual LESCs will be labelled at different stages as they cycle through active and quiescent phases, leading to the production of different length stripes. Thus, individual stem cells may only rarely produce full radial stripes if they are usually active for less time than it takes daughter TACs to move across the full radius. In the absence of intermittent quiescence, stripe lengths should increase continuously and would be less variable. Thus, the observed large variations in stripe lengths are not consistent with LESC-cc.

Few stripes were induced in most eyes labelled at 12 weeks (Fig. S2) and, although no quantitative comparisons were made, the total area of β-gal positive labelling appeared lower when stripes had fully extended by 14 weeks than when the short-lived TAC clones predominated at 2 weeks. If so, this would suggest that stem cells might label less readily than other cell types. Many more stripes per cornea and relatively more LC stripes were produced when mice were treated with tamoxifen at 4 weeks than at 12 weeks and fewer were produced in mice treated at 24 weeks. This suggests that this was a progressive age effect and not simply a difference between adults and juveniles or a technical difference in tamoxifen delivery. However, qualitative evidence for a parallel trend in the number of short-lived patches at 6 weeks (Fig. 6) indicates that the age effect is not restricted to stem cells. Further work is required to determine whether age affects the number of stem cells (or other progenitors) that can respond to tamoxifen, activate the reporter and generate stripes. Many age-related quantitative or qualitative differences in stem cells, including effects on stripe frequency, could be consistent with all four hypotheses. The higher proportion of LC stripes after treating young mice is not consistent with LESC-cc (all stripes should be LC stripes) but is consistent with LESC-iq (periods of quiescence could be less frequent or shorter in younger mice) and could also be consistent with CESC-cf and CESC-cp.

The evidence, summarised in Table 1, shows that the results are consistent with the LESC-iq hypothesis, possibly augmented by LESC loss and/or replacement. So far we have assumed that the four hypotheses are mutually exclusive but we should also consider whether the corneal epithelium could be maintained, during normal homeostasis, by a combination of LESCs and CESCs. The existence of significant numbers of both CC and LC stripes soon after stripes emerged (row 4 of Table 1) would be more consistent with a combined LESC-iq/CESC-cp hypothesis than the CESC-cp hypothesis alone. However, the evidence that long-lived clones all emerged at the periphery (row 2 of Table 1) would only be consistent with a combined hypothesis if the CESCs were predominantly located near the periphery. After 6- or 8-week chase periods, over 95% of stripes were confined to the periphery, less than 5% had their central ends in the intermediate region and none were in the centre (Fig. 4G). We cannot exclude the possibility that some peripheral LC stripes arose from CESCs right at the limbal–corneal boundary because we only checked that some LC stripes extended into the limbus itself (Fig. 5E). Thus, it remains possible that CESCs exist but this would require the ad hoc assumption that they were only in the peripheral and possibly intermediate cornea, not in the central cornea. The results favour the LESC-iq hypothesis but we cannot exclude the possibility that during normal homeostasis the corneal epithelium is maintained by LESCs with an additional contribution from some CESCs near the periphery. However, it is clear that the corneal epithelium cannot be maintained exclusively by CESCs during normal homeostasis as proposed by both CESC hypotheses (Table 1).

### 4.3 Reconciling lineage tracing and surgical transplantation results

Our study supports the results of the other two lineage tracing studies (Di Girolamo et al., 2015, Amitai-Lange et al., 2015), showing that cells labelled in the limbus colonise the corneal epithelium during normal homeostasis. As this is inconsistent with the failure of transplanted limbal cells to colonise the unwounded cornea (Majo et al., 2008), we need to explain this discrepancy. One possibility is that the transplanted cells failed to colonise the cornea because the surgical procedure perturbed normal homeostasis. For example, surgical sutures in the cornea can induce corneal vascularisation (Kather and Kroll, 2014) and this might interfere with corneal epithelial cell movement (Zhao et al., 2011). If corneal homeostasis were compromised, the transplantation experiment would not be a suitable test of whether LESCs contribute cells to the corneal epithelium during normal homeostasis.

### 4.4 Stem cell quiescence and the LESC hypothesis

Many types of stem cells are quiescent and it has been suggested that, although they have exited the cell cycle, they are not dormant but poised for activation (Cheung and Rando, 2013). However, recent work suggests that two levels of stem cell quiescence exist: a deep quiescence and a poised or alert quiescence (Rodgers et al., 2014, Rumman et al., 2015). Although it is not known whether this applies to LESCs, these observations invite speculation. For example, if LESCs existed in three interchangeable states, tamoxifen treatment might produce stripes immediately from active LESCs or after a delay from poised quiescent LESCs but deep quiescent LESCs would not produce stripes during normal homeostasis if they were only activated in response to wounding. The presence of CC stripes would then suggest that, during normal homeostasis, some LESCs cycled between active and poised quiescent states. If relative frequencies of the three stem cell states varied with age, the increase in LC stripes and overall labelling frequency produced by treating young mice with tamoxifen could be explained if young mice have more active LESCs and fewer deep quiescent LESCs than older mice. This would predict compensatory age-related changes in TAC proliferation and/or cell loss to avoid disturbing homeostasis. This does not, however, explain the age-related differences in numbers of short-lived TAC clones.

### 4.5 Conclusions

Using an unbiased lineage tracing system that labels all cell types, we showed that labelled clones appeared at the periphery and extended centripetally as radial stripes but we found no evidence that long-lived clones also arose in the central corneal epithelium, as proposed by all versions of the CESC hypothesis. These results are consistent with the LESC hypothesis if LESCs cycle through intermittent periods of activity and quiescence, each lasting several weeks. Loss of labelled LESCs or replacement by unlabelled LESCs (stochastic neutral drift) may also occur. Detailed cell cycle analysis may be useful for testing whether LESCs show intermittent quiescence, as our study predicts, and for determining whether LESCs exist in different quiescent states.

The following are the supplementary data related to this article.

Supplementary Fig. S1 β-gal staining of corneas and conjunctivas with possible leaky ROSA26R-*LacZ* reporter expression.
(A–H) Comparison of β-gal staining in corneas and conjunctivas of five eyes with most extensive radial stripes (49R, 39L, 39R, 161L and 161R) and two eyes with more typical labelling patterns (49L and 40R). (A, B) Eyes 49L and 49R from the same mouse after a 12-week chase. Eye 49L had two stripes and small patches in the conjunctiva but the contralateral eye, 49R, had several long corneal stripes and medium sized conjunctival patches. (C, D) Eyes 39L and 39R from the same mouse, after a 14-week chase, both had long stripes and medium sized conjunctival patches (see Fig. 2L for another view of eye 39L). (E–G) Eyes 161L and 161R from the same mouse, after an 18-week chase. Both eyes had many wide stripes spanning the corneal radius and both medium and large conjunctival patches. The large number of wide corneal stripes and the large conjunctival patches in eyes 161L and 161R, makes it likely that labelling occurred during development. The staining pattern appears similar to that reported for chimeras and X-inactivation mosaics (Collinson et al., 2002, Mort et al., 2009), where labelled stem cells are likely to be arranged in clonally related groups. (H) Eye 40R after a 14-week chase, showed a more typical pattern of corneal stripes and is included for comparison. Most conjunctival patches were small but there were several medium sized patches. Arrows in B, D and F show that stripes formed a central whorl. Photograph B is shown in Fig. 2J but is also included here to allow comparisons among the five eyes with long stripes. (I–N) The positions and lengths of β-gal positive stripes and radially aligned unstained areas are shown relative to the limbus as described in the legend to Fig. 3. The top horizontal line in each graph indicates the mean radius for the group and the other two lines divide the radius into equal lengths to define peripheral (P), intermediate (I) and central (C) regions. These regions are arranged concentrically in the intact eye. LC stripes (with one end at the limbus) are shown separately from CC stripes, which do not include the limbus, and stripes are ordered by position. In bar charts I, K and M, β-gal positive stripes from eyes 49R, 39L, 39R, 161L and 161R, are shown as a paler blue colour than the stripes from other eyes. (I, J) Distributions of radial stripes after 12 weeks chase, including (I) or excluding (J) eye 49R, shown in B. (K, L) Distributions of radial stripes after 14 weeks chase, including (K) or excluding (L) eyes 39L and 39R, shown in C and D. The asterisks in K indicate two stripes that curved into a central whorl and were longer than 1800 μm. (M, N) Distributions of radial stripes after 18 weeks chase, including (M) or excluding (N) eyes 161L and 161R, shown in E–G. These eyes had the longest and widest stripes in the group and most stripes were LC stripes, which would be predicted if stem cells were arranged in clonally related groups. (If a β-gal positive stem cell enters quiescence, its stripe is more likely to be continued by a neighbouring β-gal positive stem cell than a neighbouring β-gal negative stem cell if clusters of neighbouring stem cells are clonally relatEd.) Abbreviations: L, left; R, right.

Supplementary Fig. S2 Variation in β-gal positive stripe numbers per cornea after labelling at 12 weeks.
Results were analysed separately with and without data from eyes 39L, 39R and 49R, illustrated in Supplementary Fig. S1, as it was uncertain whether they had any stripes that arose by leaky expression. In each case, data from mouse 161 were excluded, as some stripes were likely to have arisen by leaky expression during development. (A, B) The number of stripes per cornea varied and was positively correlated between left and right eyes regardless of whether mice 39 and 49 were included (A) or excluded (B), as shown in the figure. Different chase times and genders are indicated but were not analysed separately. The results shown in A and B were analysed again after omitting eyes with little or no staining, which could have included technical failures and might have increased the correlation but left and right eyes remained correlated (r_s_ = 0.5311; *P* < 0.0001 for A and r_s_ = 0.5303; *P* = 0.0002 for B). (C–F) Regardless of whether eyes 39L, 39R and 49R were included (C, E) or excluded (D, F), the number of stripes per cornea did not differ significantly among chase times for either left (C, D) or right (E, F) eyes, which were analysed separately as they were not independent (as shown in A, B). Data were analysed by the Kruskal–Wallis (KW) test and Dunn's multiple comparison post-test. Males and females are shown in the figures but were not analysed separately.

Supplementary Fig. S3 Effects of chase time on β-gal positive stripes induced at 12 weeks, excluding eyes 49R, 39L and 39R.
(A-G) Results of the type of analyses shown in Fig. 4 but excluding eyes 49R, 39L and 39R (as well as 161L and 161R), shown in Supplementary Fig. S1. (A) Comparisons of the distance between the limbus and the central end of β-gal stripes by Kruskal–Wallis (KW) test and Dunn's multiple comparison tests. (Shared letters above the box and whisker plots indicate no significant difference; for other comparisons, *P* < 0.05.) (B) Fisher's exact tests (asterisks) show that a higher percentage of stripes have their central end > 500 μm from the limbus after chase times of 10–20 weeks than for 6 and 8 weeks combined. (C) Fisher's exact tests (asterisks) show that a higher percentage of stripes have their central end > 1000 μm from the limbus after chase times of 14–20 weeks than for 6–12 weeks combined. (D) Comparisons of the distance between the limbus and the central end of the most central 10% of the β-gal stripes. Linear regression showed stripes extended centripetally between 6 and 14 weeks (42–98 days). (E) The percentage of stripes that have one end at the limbus (LC stripes) varied among chase times but did not increase with chase time. (There was a weak trend to decrease with chase time.) (F) Comparisons of the distance between the limbus and the peripheral end of β-gal CC stripes (which do not include the limbus) by Kruskal–Wallis test and Dunn's multiple comparison tests. (Shared letters above the box and whisker plots indicate no significant difference; for other comparisons *P* < 0.05.) Distances increased after 8 weeks. (G) Comparison of the percentage of the central ends of β-gal stripes, in the peripheral (P), intermediate (I) and central (C) regions of the cornea, after different chase times, as described in the text and legend to Fig. 4. For each chase time (6–20 weeks) and the K5 group (adult *KRT5LacZ/−* mosaic mice; Douvaras et al., 2012), the observed P:I:C distributions differed significantly from the expected proportions (Exp) by goodness of fit chi-square test. After chase times of 14–20 weeks, the P:I:C distributions did not differ significantly from the adult K5 transgenic eyes (Fisher's exact tests). In the box and whisker plots the whiskers show the minimum and maximum values. The number of stripes analysed at each chase time is shown within the boxes in A. NS, not significant; * *P* < 0.05; ** *P* < 0.01; *** *P* < 0.001; **** *P* < 0.0001.

Supplementary Fig. S4 Estimation of number of stem cells per cornea from β-gal positive stripes induced at 12 weeks.
(A, B) The distribution of LC stripes widths (measured at the cornea–limbal boundary) in eyes of mice injected with tamoxifen at 12 weeks and chased for 6–20 weeks (including eyes 49R, 39L and 39R but excluding eyes 161L and 161R, shown in Supplementary Fig. S1) is shown for all LC stripes (A) and 20–150 μm wide stripes (B). In B one outlier that was < 20 μm and six outliers that were > 150 μm wide were excluded, as the wider stripes are likely to be derived from more than one stem cell. The peak stripe width was 35–40 μm and the overall mean circumference was 10,365 μm, so 259–296 stripes would fit around the limbal circumference. This provides an estimate of the number of stem cells that maintain the corneal epithelium but assumes they are evenly distributed around the circumference. (C, D) The distribution of circumference / stripe width ratios at the cornea–limbal boundary (using separate circumference measurements for each eye) is shown for all LC stripes (C) and for 20–150 μm wide stripes (D). The peak circumference/stripe width ratio provides another estimate the number of stripes that would fit around the limbal circumference but the peak estimates were rather broad (175–300). (E) Comparison of stripe widths (for 20–150 μm wide stripes) among chase times of 6–20 weeks after tamoxifen injection at 12 weeks by Kruskal–Wallis (KW) tests and Dunn's multiple comparison tests. (Shared letters indicate no significant difference; for other comparisons, *P* < 0.05.) This also tests for differences in stripe widths among ages because, for this series, age = chase time + 12 weeks. (F) Comparison of circumference/stripe width ratios (for 20–150 μm wide stripes) among chase times of 6–20 weeks after tamoxifen injection at 12 weeks by 1-way analysis of variance (ANOVA) and Tukey's multiple comparison tests. (Shared letters indicate no significant difference; for other comparisons, *P* < 0.05.) In the box and whisker plots the whiskers show the minimum and maximum values.

Supplementary Fig. S5 Numbers and widths of β-gal positive stripes induced at different ages.
(A, B) The number of stripes per cornea was positively correlated between left and right eyes for mice treated with tamoxifen at 4 weeks (A) but not for the smaller sample treated at 24 weeks (B). (For mice treated at 24 weeks, only 13/22 eyes displayed stripes; Fig. 7K.) Pearson's correlation coefficients are shown in the figure. Different chase times and genders are indicated but were not analysed separately. (C–F) The distributions of LC stripes widths, measured at the cornea–limbal boundary (C, E) and the circumference/stripe width ratios (D, F) in eyes of mice injected with tamoxifen at 4 weeks and chased for 6 (C, D) or 12 (E, F) weeks. The analysis method is described in the legend to Supplementary Fig. S4.

Supplementary material: legends for Supplementary Figs. S1-S5.

## Figures and Tables

**Table 1:** Consistency of lineage analysis results with alternative hypotheses.

Lineage tracing result	Consistent with hypothesis?			
CESC-cf	CESC-cp	LESC-cc	LESC-iq
Most labelled clones were short lived	+	+	+	+
Long-lived clones all emerged at the periphery	−	−	+	+
Stripes extended towards the centre	−	+	+	+
Significant numbers of both CC and LC stripes were present soon after stripes emerged	±	±	−	+
The distance between the limbus and CC stripes did not decrease with chase time	−	+	−*	+
The frequency of LC stripes labelled at 12 weeks did not increase and was 35–55% at all chase times	−	+	−*	+
Few stripes extended completely to the centre even after long chase times	+	+	+	+
Some stripes were discontinuous	+	+	+	+
The distance of the central stripe end from the limbus varied widely at all chase times	+	+	−	+
Stripe frequency varied with age at labelling.	+	+	+	+
The proportion of LC stripes varied with age at labelling	+	+	−*	+

+, results are consistent with hypothesis.

±, results are not expected but could be consistent with hypothesis if specific assumptions are made.

−, results are not consistent with hypothesis.

*, inconsistent with LESC-cc hypothesis because it predicts all stripes should be LC stripes.

Abbreviations:

CESC-cf Corneal epithelial stem cell hypothesis with centrifugal movement.

CESC-cp Corneal epithelial stem cell hypothesis with centripetal movement.

LESC-cc Limbal epithelial stem cell hypothesis with continuously slow cycling stem cells.

LESC-iq Limbal epithelial stem cell hypothesis with intermittent quiescence.
