# Supplementary figures and images for: Lineage tracing in the adult mouse corneal epithelium supports the limbal epithelial stem cell hypothesis with intermittent periods of stem cell quiescence
Source: Stem Cell Res. 2015 Nov;15(3):665–77. doi: 10.1016/j.scr.2015.10.016 (PMC4686565; doi:10.1016/j.scr.2015.10.016)

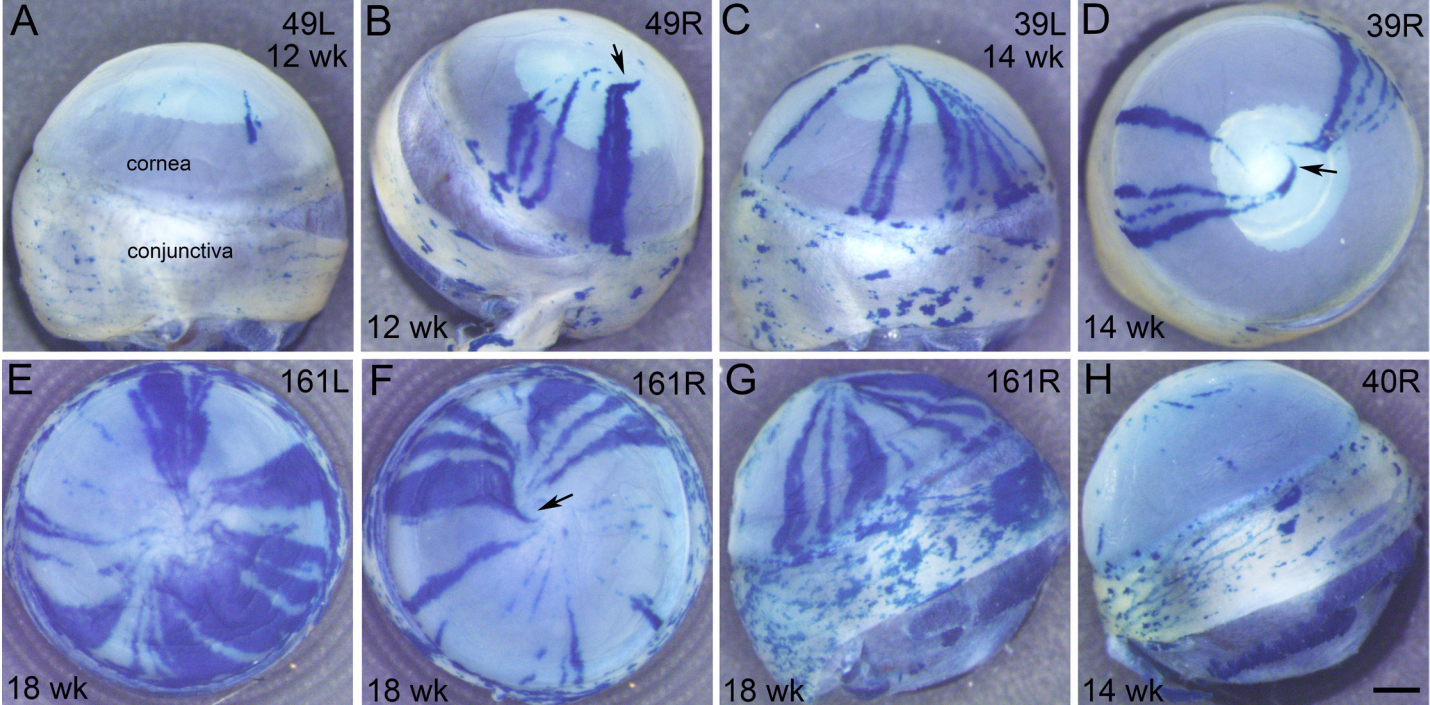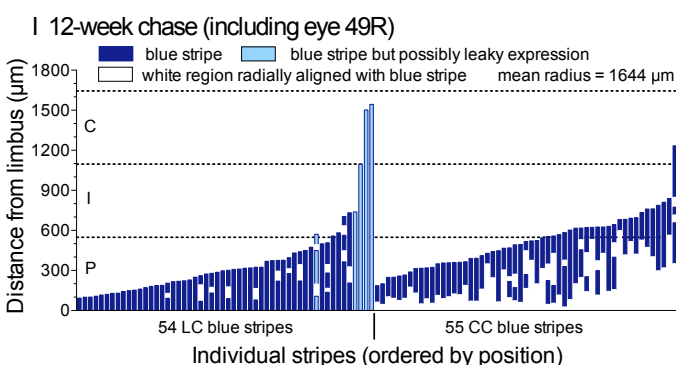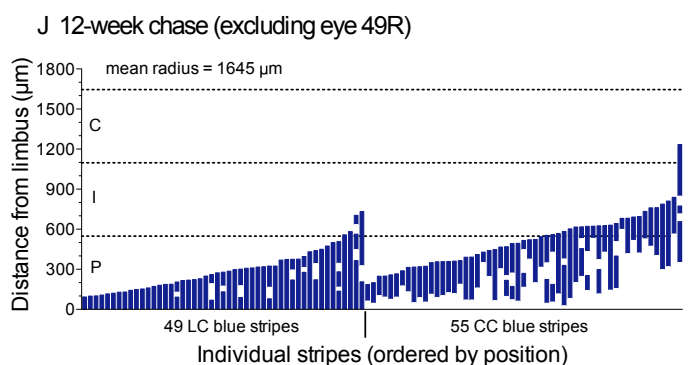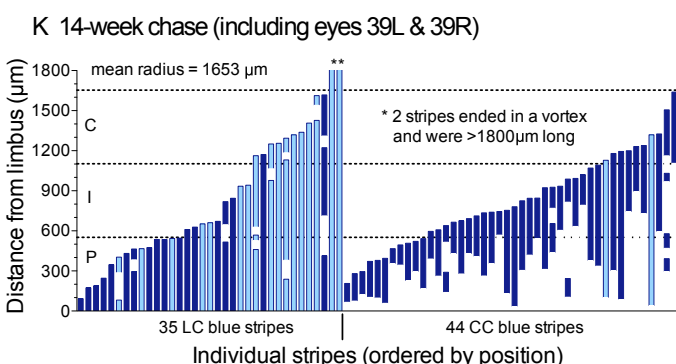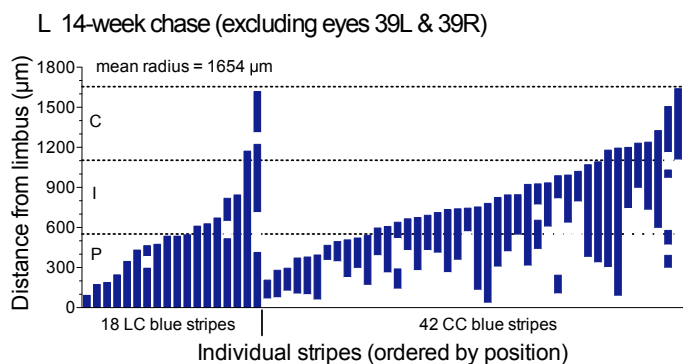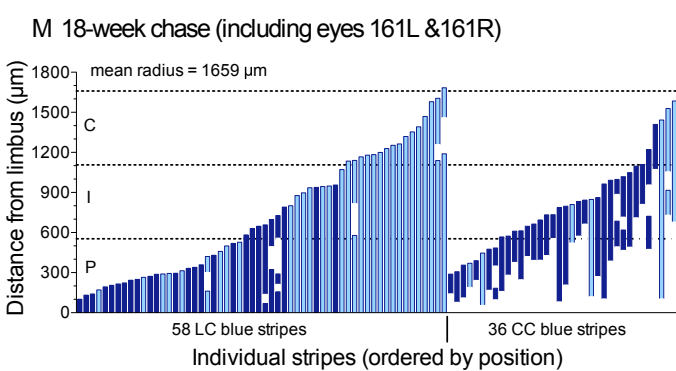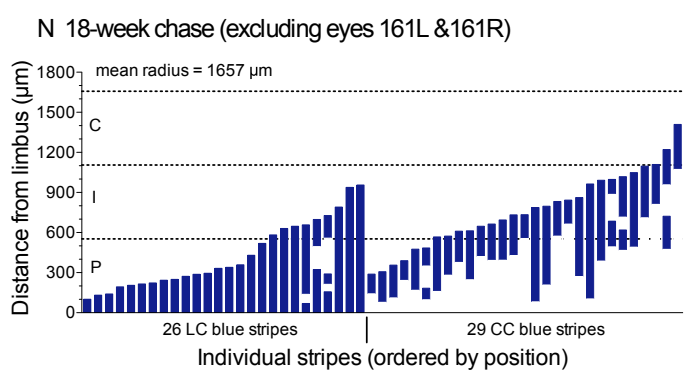

Supplement: Supplementary Fig. S1 — β-gal staining of corneas and conjunctivas with possible leaky ROSA26R-LacZ reporter expression. (A–H) Comparison of β-gal staining in corneas and conjunctivas of five eyes with most extensive radial stripes (49R, 39L, 39R, 161L and 161R) and two eyes with more typical labelling patterns (49L and 40R). (A, B) Eyes 49L and 49R from the same mouse after a 12-week chase. Eye 49L had two stripes and small patches in the conjunctiva but the contralateral eye, 49R, had several long corneal stripes and medium sized conjunctival patches. (C, D) Eyes 39L and 39R from the same mouse, after a 14-week chase, both had long stripes and medium sized conjunctival patches (see Fig. 2L for another view of eye 39L). (E–G) Eyes 161L and 161R from the same mouse, after an 18-week chase. Both eyes had many wide stripes spanning the corneal radius and both medium and large conjunctival patches. The large number of wide corneal stripes and the large conjunctival patches in eyes 161L and 161R, makes it likely that labelling occurred during development. The staining pattern appears similar to that reported for chimeras and X-inactivation mosaics (Collinson et al., 2002, Mort et al., 2009), where labelled stem cells are likely to be arranged in clonally related groups. (H) Eye 40R after a 14-week chase, showed a more typical pattern of corneal stripes and is included for comparison. Most conjunctival patches were small but there were several medium sized patches. Arrows in B, D and F show that stripes formed a central whorl. Photograph B is shown in Fig. 2J but is also included here to allow comparisons among the five eyes with long stripes. (I–N) The positions and lengths of β-gal positive stripes and radially aligned unstained areas are shown relative to the limbus as described in the legend to Fig. 3. The top horizontal line in each graph indicates the mean radius for the group and the other two lines divide the radius into equal lengths to define peripheral (P), intermediate (I) and centr [file mmc1.pdf]

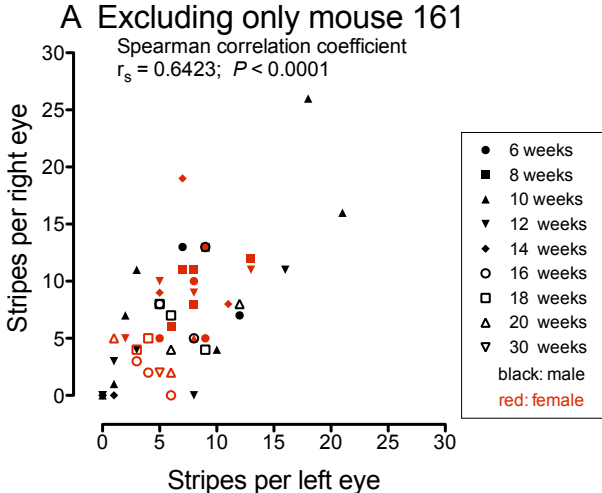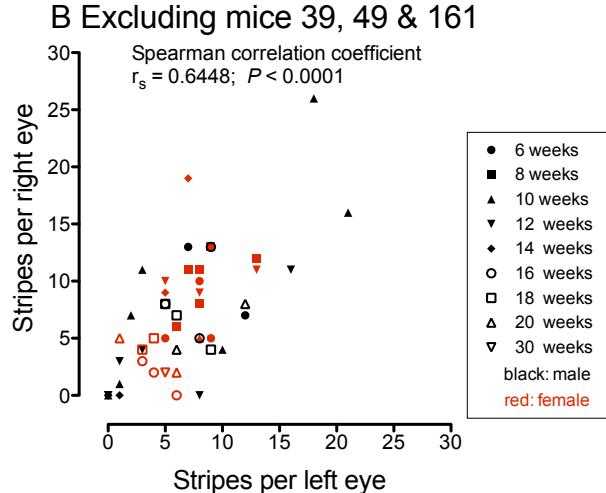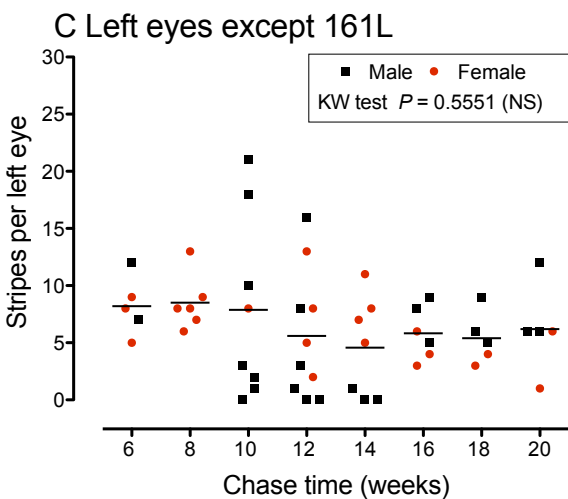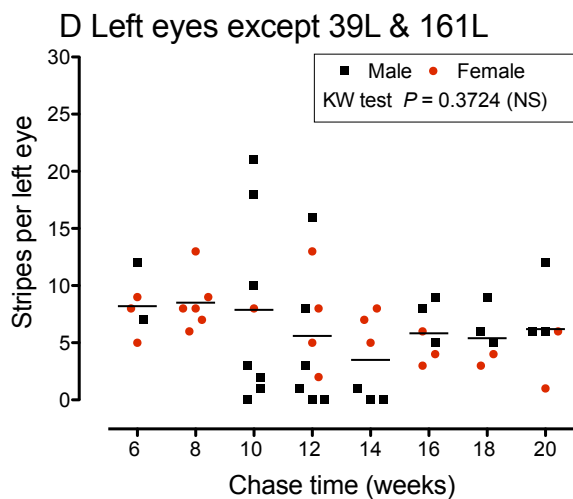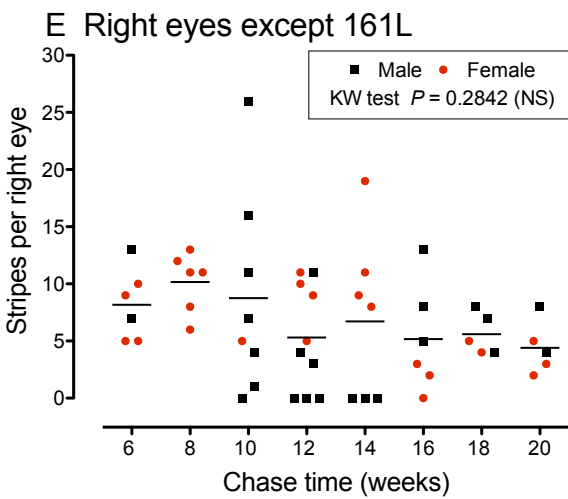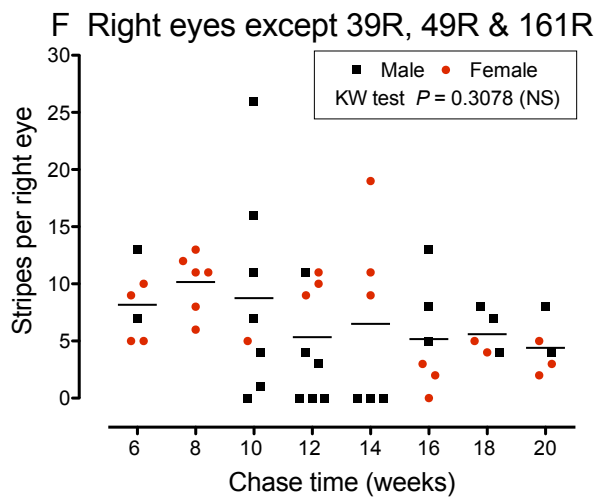

Supplement: Supplementary Fig. S2 — Variation in β-gal positive stripe numbers per cornea after labelling at 12 weeks. Results were analysed separately with and without data from eyes 39L, 39R and 49R, illustrated in Supplementary Fig. S1, as it was uncertain whether they had any stripes that arose by leaky expression. In each case, data from mouse 161 were excluded, as some stripes were likely to have arisen by leaky expression during development. (A, B) The number of stripes per cornea varied and was positively correlated between left and right eyes regardless of whether mice 39 and 49 were included (A) or excluded (B), as shown in the figure. Different chase times and genders are indicated but were not analysed separately. The results shown in A and B were analysed again after omitting eyes with little or no staining, which could have included technical failures and might have increased the correlation but left and right eyes remained correlated (rs = 0.5311; P < 0.0001 for A and rs = 0.5303; P = 0.0002 for B). (C–F) Regardless of whether eyes 39L, 39R and 49R were included (C, E) or excluded (D, F), the number of stripes per cornea did not differ significantly among chase times for either left (C, D) or right (E, F) eyes, which were analysed separately as they were not independent (as shown in A, B). Data were analysed by the Kruskal–Wallis (KW) test and Dunn's multiple comparison post-test. Males and females are shown in the figures but were not analysed separately. [file mmc2.pdf]

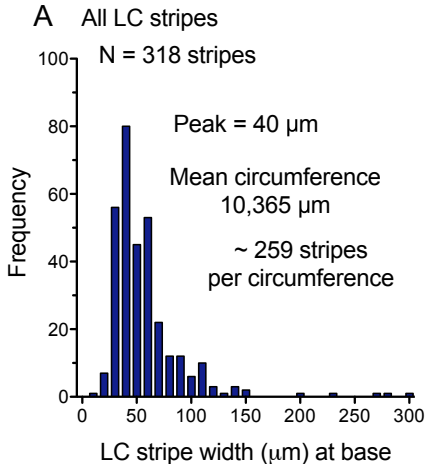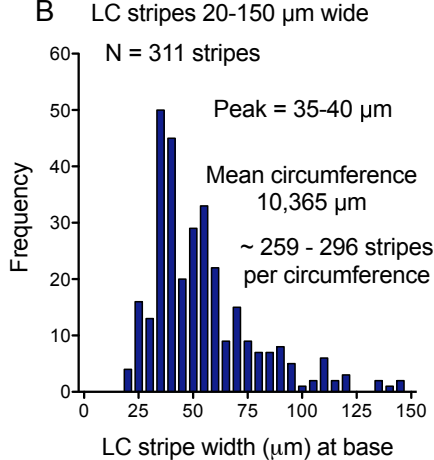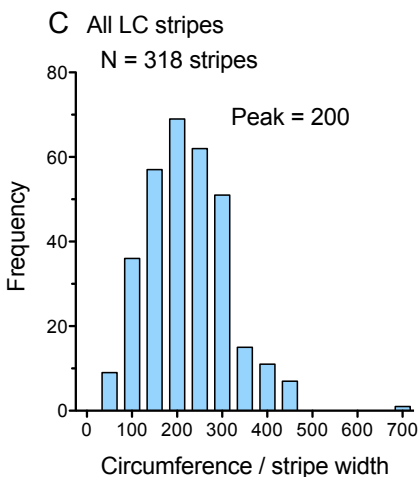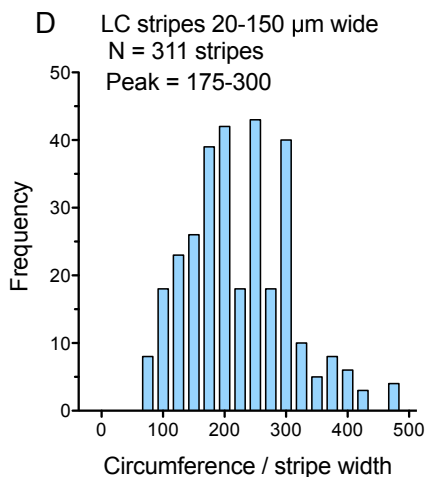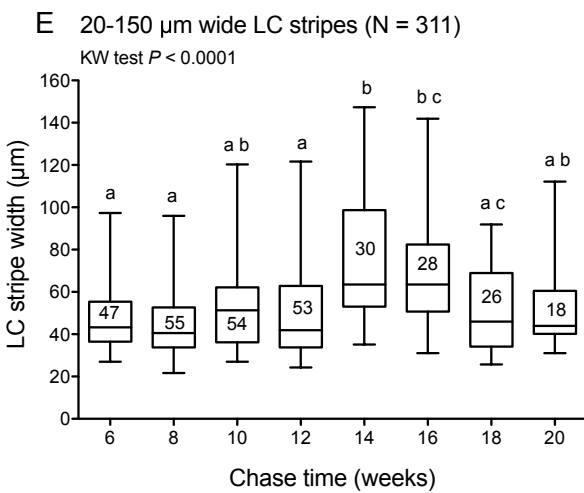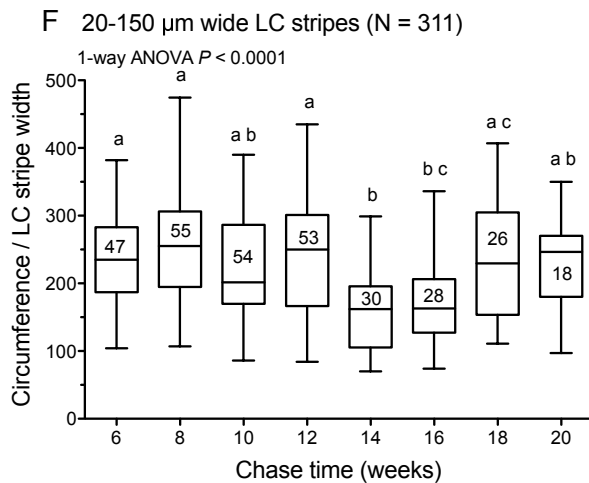

Supplement: Supplementary Fig. S4 — Estimation of number of stem cells per cornea from β-gal positive stripes induced at 12 weeks. (A, B) The distribution of LC stripes widths (measured at the cornea–limbal boundary) in eyes of mice injected with tamoxifen at 12 weeks and chased for 6–20 weeks (including eyes 49R, 39L and 39R but excluding eyes 161L and 161R, shown in Supplementary Fig. S1) is shown for all LC stripes (A) and 20–150 μm wide stripes (B). In B one outlier that was < 20 μm and six outliers that were > 150 μm wide were excluded, as the wider stripes are likely to be derived from more than one stem cell. The peak stripe width was 35–40 μm and the overall mean circumference was 10,365 μm, so 259–296 stripes would fit around the limbal circumference. This provides an estimate of the number of stem cells that maintain the corneal epithelium but assumes they are evenly distributed around the circumference. (C, D) The distribution of circumference / stripe width ratios at the cornea–limbal boundary (using separate circumference measurements for each eye) is shown for all LC stripes (C) and for 20–150 μm wide stripes (D). The peak circumference/stripe width ratio provides another estimate the number of stripes that would fit around the limbal circumference but the peak estimates were rather broad (175–300). (E) Comparison of stripe widths (for 20–150 μm wide stripes) among chase times of 6–20 weeks after tamoxifen injection at 12 weeks by Kruskal–Wallis (KW) tests and Dunn's multiple comparison tests. (Shared letters indicate no significant difference; for other comparisons, P < 0.05.) This also tests for differences in stripe widths among ages because, for this series, age = chase time + 12 weeks. (F) Comparison of circumference/stripe width ratios (for 20–150 μm wide stripes) among chase times of 6–20 weeks after tamoxifen injection at 12 weeks by 1-way analysis of variance (ANOVA) and Tukey's multiple comparison tests. (Shared letters indicate no significant difference; for other comparisons, [file mmc4.pdf]

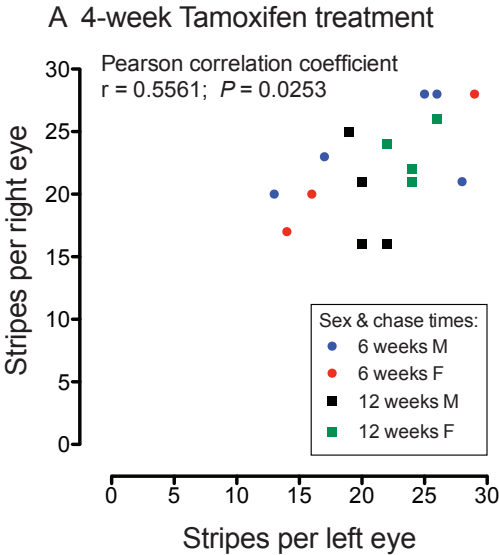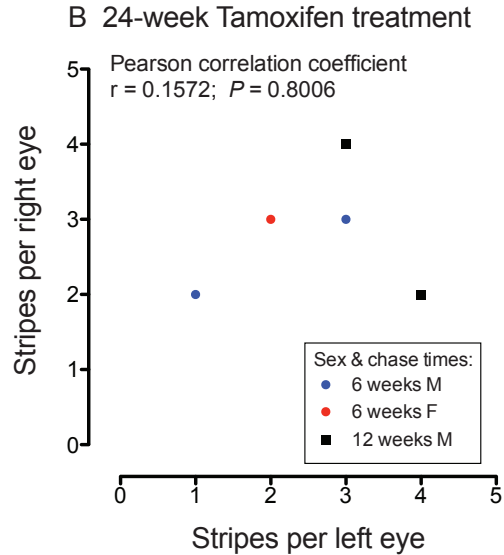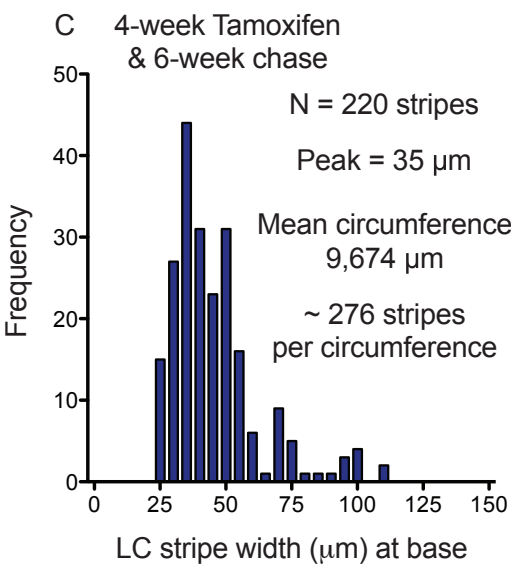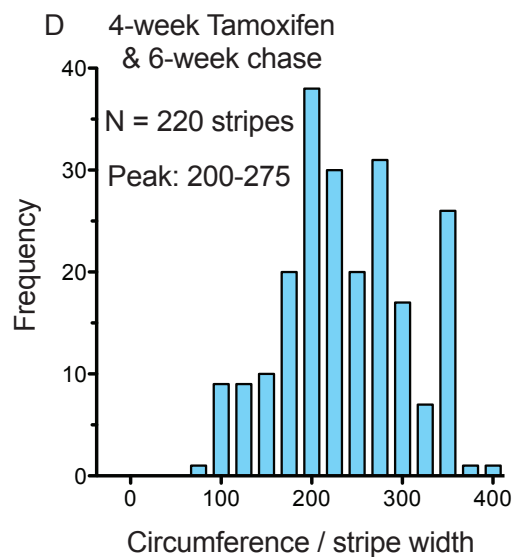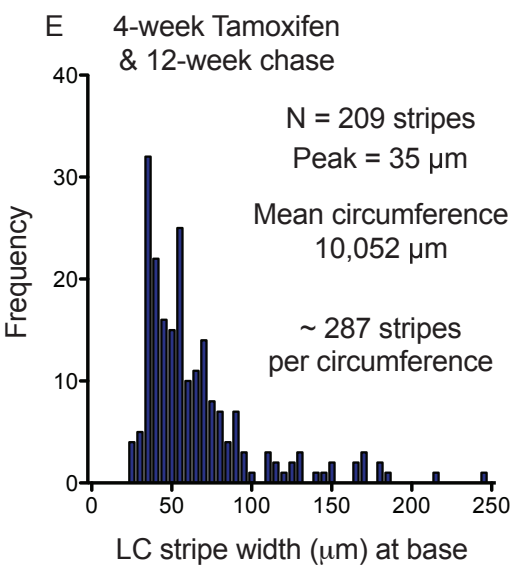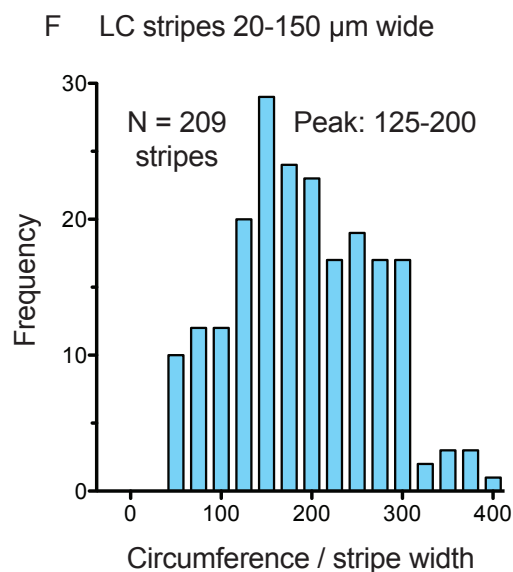

Supplement: Supplementary Fig. S5 — Numbers and widths of β-gal positive stripes induced at different ages. (A, B) The number of stripes per cornea was positively correlated between left and right eyes for mice treated with tamoxifen at 4 weeks (A) but not for the smaller sample treated at 24 weeks (B). (For mice treated at 24 weeks, only 13/22 eyes displayed stripes; Fig. 7K.) Pearson's correlation coefficients are shown in the figure. Different chase times and genders are indicated but were not analysed separately. (C–F) The distributions of LC stripes widths, measured at the cornea–limbal boundary (C, E) and the circumference/stripe width ratios (D, F) in eyes of mice injected with tamoxifen at 4 weeks and chased for 6 (C, D) or 12 (E, F) weeks. The analysis method is described in the legend to Supplementary Fig. S4. [file mmc5.pdf]
