# Supplementary material for: Lineage tracing in the adult mouse corneal epithelium supports the limbal epithelial stem cell hypothesis with intermittent periods of stem cell quiescence
Source: Stem Cell Res. 2015 Nov;15(3):665–77. doi: 10.1016/j.scr.2015.10.016 (PMC4686565; doi:10.1016/j.scr.2015.10.016)
Supplement: Supplementary Fig. S3 — Effects of chase time on β-gal positive stripes induced at 12 weeks, excluding eyes 49R, 39L and 39R. (A-G) Results of the type of analyses shown in Fig. 4 but excluding eyes 49R, 39L and 39R (as well as 161L and 161R), shown in Supplementary Fig. S1. (A) Comparisons of the distance between the limbus and the central end of β-gal stripes by Kruskal–Wallis (KW) test and Dunn's multiple comparison tests. (Shared letters above the box and whisker plots indicate no significant difference; for other comparisons, P < 0.05.) (B) Fisher's exact tests (asterisks) show that a higher percentage of stripes have their central end > 500 μm from the limbus after chase times of 10–20 weeks than for 6 and 8 weeks combined. (C) Fisher's exact tests (asterisks) show that a higher percentage of stripes have their central end > 1000 μm from the limbus after chase times of 14–20 weeks than for 6–12 weeks combined. (D) Comparisons of the distance between the limbus and the central end of the most central 10% of the β-gal stripes. Linear regression showed stripes extended centripetally between 6 and 14 weeks (42–98 days). (E) The percentage of stripes that have one end at the limbus (LC stripes) varied among chase times but did not increase with chase time. (There was a weak trend to decrease with chase time.) (F) Comparisons of the distance between the limbus and the peripheral end of β-gal CC stripes (which do not include the limbus) by Kruskal–Wallis test and Dunn's multiple comparison tests. (Shared letters above the box and whisker plots indicate no significant difference; for other comparisons P < 0.05.) Distances increased after 8 weeks. (G) Comparison of the percentage of the central ends of β-gal stripes, in the peripheral (P), intermediate (I) and central (C) regions of the cornea, after different chase times, as described in the text and legend to Fig. 4. For each chase time (6–20 weeks) and the K5 group (adult KRT5LacZ/− mosaic mice; Douvaras et al., 2012), the observed P:I:C d [file mmc3.pdf]

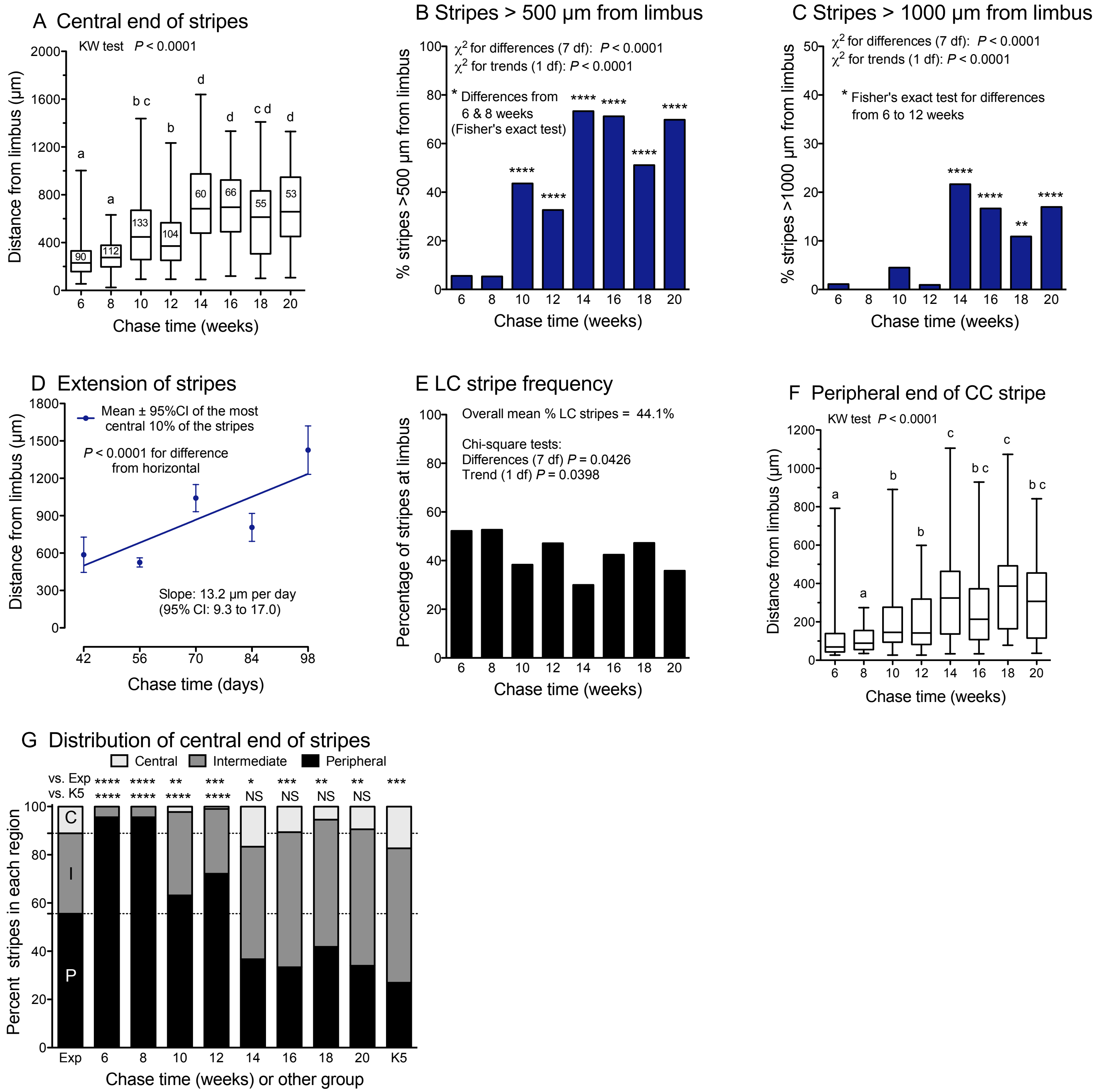

A Central end of stripes
KW test P < 0.0001
Distance from limbus (µm)
Chase time (weeks)
B Stripes > 500 µm from limbus
χ2 for differences (7 df): P < 0.0001
χ2 for trends (1 df): P < 0.0001
* Differences from 6 & 8 weeks (Fisher's exact test)
% stripes >500 µm from limbus
Chase time (weeks)
C Stripes > 1000 µm from limbus
χ2 for differences (7 df): P < 0.0001
χ2 for trends (1 df): P < 0.0001
* Fisher's exact test for differences from 6 to 12 weeks
% stripes >1000 µm from limbus
Chase time (weeks)
D Extension of stripes
Mean ± 95%CI of the most central 10% of the stripes
P < 0.0001 for difference from horizontal
Slope: 13.2 µm per day (95% CI: 9.3 to 17.0)
Distance from limbus (µm)
Chase time (days)
E LC stripe frequency
Overall mean % LC stripes = 44.1%
Chi-square tests:
Differences (7 df) P = 0.0426
Trend (1 df) P = 0.0398
Percentage of stripes at limbus
Chase time (weeks)
F Peripheral end of CC stripe
KW test P < 0.0001
Distance from limbus (µm)
Chase time (weeks)
G Distribution of central end of stripes
Central
Intermediate
Peripheral
vs. Exp
vs. K5
Percent stripes in each region
Chase time (weeks) or other group
