# Supplementary material for: Lineage tracing in the adult mouse corneal epithelium supports the limbal epithelial stem cell hypothesis with intermittent periods of stem cell quiescence
Source: Stem Cell Res. 2015 Nov;15(3):665–77. doi: 10.1016/j.scr.2015.10.016 (PMC4686565; doi:10.1016/j.scr.2015.10.016)
Supplement: Supplementary file 6 — Supplementary material: legends for Supplementary Figs. S1-S5. [file mmc6.pdf]

Lineage tracing in the adult mouse corneal epithelium supports the limbal epithelial stem cell hypothesis with intermittent periods of stem cell quiescence

Natalie J. Dorà, Robert E. Hill, J. Martin Collinson & John D. West

---

SUPPLEMENTARY FIGURE LEGENDS

Supplementary Fig S1. β-gal staining of corneas and conjunctivas with possible leaky ROSA26R-*LacZ* reporter expression

(A-H) Comparison of β-gal staining in corneas and conjunctivas of five eyes with most extensive radial stripes (49R, 39L, 39R, 161L and 161R) and two eyes with more typical labelling patterns (49L and 40R). **(A, B)** Eyes 49L and 49R from the same mouse after a 12-week chase. Eye 49L had two stripes and small patches in the conjunctiva but the contralateral eye, 49R, had several long corneal stripes and medium sized conjunctival patches (**C, D**) Eyes 39L and 39R from the same mouse, after a 14-week chase, both had long stripes and medium sized conjunctival patches (see Fig. 2L for another view of eye 39L). (**E-G**) Eyes 161L and 161R from the same mouse, after an 18-week chase. Both eyes had many wide stripes spanning the corneal radius and both medium and large conjunctival patches. The large number of wide corneal stripes and the large conjunctival patches in eyes 161L and 161R, makes it likely that labelling occurred during development. The staining pattern appears similar to that reported for chimaeras and X-inactivation mosaics (Collinson et al 2002; Mort et al 2009), where labelled stem cells are likely to be arranged in clonally related groups. (**H**) Eye 40R after a 14-week chase, showed a more typical pattern of corneal stripes and is included for comparison. Most conjunctival patches were small but there were several medium sized patches. Arrows in B, D and F show that stripes formed a central whorl. Photograph B is shown in Fig. 2J but is also included here to allow comparisons among the five eyes with long stripes. (**I-N**) The positions and lengths of β-gal positive stripes and radially aligned unstained areas are shown relative to the limbus as described in the legend to Fig. 3. The top horizontal line in each graph indicates the mean radius for the group and the other two lines divide the radius into equal lengths to define peripheral (P), intermediate (I) and central (C) regions. These regions are arranged concentrically in the intact eye. LC stripes (with one end at the limbus) are shown separately from CC stripes, which do not include the limbus, and stripes are ordered by position. In bar charts I, K and M, β-gal positive stripes from eyes 49R, 39L, 39R, 161L and 161R, are shown as a paler blue colour than the stripes from other eyes. (**I, J**) Distributions of radial stripes after 12 weeks chase, including (I) or excluding (J) eye 49R, shown in B. (**K, L**) Distributions of radial stripes after 14 weeks chase, including (K) or excluding (L) eyes 39L and 39R, shown in C and D. The asterisks in K indicate two stripes that curved into a central whorl and

were longer than 1800 μm. (**M, N**) Distributions of radial stripes after 18 weeks chase, including (M) or excluding (N) eyes 161L and 161R, shown in E-G. These eyes had the longest and widest stripes in the group and most stripes were LC stripes, which would be predicted if stem cells were arranged in clonally related groups. (If a β-gal positive stem cell enters quiescence, its stripe is more likely to be continued by a neighbouring β-gal positive stem cell than a neighbouring β-gal negative stem cell if clusters of neighbouring stem cells are clonally related.) Abbreviations: L, left; R, right.

Supplementary Fig. S2. Variation in β-gal positive stripe numbers per cornea after labelling at 12 weeks

Results were analysed separately with and without data from eyes 39L, 39R and 49R, illustrated in Supplementary Fig. S1, as it was uncertain whether they had any stripes that arose by leaky expression. In each case, data from mouse 161 were excluded, as some stripes were likely to have arisen by leaky expression during development. **(A, B)** The number of stripes per cornea varied and was positively correlated between left and right eyes regardless of whether mice 39 and 49 were included (A) or excluded (B), as shown in the figure. Different chase times and genders are indicated but were not analysed separately. The results shown in A and B were analysed again after omitting eyes with little or no staining, which could have included technical failures and might have increased the correlation but left and right eyes remained correlated ($r_s = 0.5311$; $P < 0.0001$ for A and $r_s = 0.5303$; $P = 0.0002$ for B). **(C-F)** Regardless of whether eyes 39L, 39R and 49R were included (C, E) or excluded (D, F), the number of stripes per cornea did not differ significantly among chase times for either left (C, D) or right (E, F) eyes, which were analysed separately as they were not independent (as shown in A, B). Data were analysed by the Kruskal-Wallis (KW) test and Dunn's multiple comparison post-test. Males and females are shown in the figures but were not analysed separately.

Supplementary Fig S3. Effects of chase time on β-gal positive stripes induced at 12 weeks, excluding eyes 49R, 39L and 39R

(A-G) Results of the type of analyses shown in Fig. 4 but excluding eyes 49R, 39L and 39R (as well as 161L and 161R), shown in Supplementary Fig S1. **(A)** Comparisons of the distance between the limbus and the central end of β-gal stripes by Kruskal-Wallis (KW) test and Dunn's multiple comparison tests. (Shared letters above the box and whisker plots indicate no significant difference; for other comparisons, $P < 0.05$.) **(B)** Fisher's exact tests (asterisks) show that a higher percentage of stripes have their central end > 500 μm from the limbus after chase times of 10-20 weeks than for 6 and 8 weeks combined. **(C)** Fisher's exact tests (asterisks) show that a higher percentage of stripes have their central end > 1000 μm from the limbus after chase times of 14-20 weeks than for 6-12 weeks

combined. **(D)** Comparisons of the distance between the limbus and the central end of the most central 10% of the β-gal stripes. Linear regression showed stripes extended centripetally between 6 and 14 weeks (42-98 days). **(E)** The percentage of stripes that have one end at the limbus (LC stripes) varied among chase times but did not increase with chase time. (There was a weak trend to decrease with chase time.) **(F)** Comparisons of the distance between the limbus and the peripheral end of β-gal CC stripes (which do not include the limbus) by Kruskal-Wallis test and Dunn's multiple comparison tests. (Shared letters above the box and whisker plots indicate no significant difference; for other comparisons $P <$ 0.05.) Distances increased after 8 weeks. (**G**) Comparison of the percentage of the central ends of β-gal stripes, in the peripheral (P), intermediate (I) and central (C) regions of the cornea, after different chase times, as described in the text and legend to Fig. 4. For each chase time (6-20 weeks) and the K5 group (adult $KRT5^{LacZ/-}$ mosaic mice; Douvaras et al 2012), the observed P : I : C distributions differed significantly from the expected proportions (Exp) by goodness of fit chi-square test. After chase times of 14-20 weeks, the P : I : C distributions did not differ significantly from the adult K5 transgenic eyes (Fisher's exact tests). In the box and whisker plots the whiskers show the minimum and maximum values. The number of stripes analysed at each chase time is shown within the boxes in A. NS, not significant; * $P < 0.05$; ** $P < 0.01$; *** $P < 0.001$; **** $P < 0.0001$.

Supplementary Fig S4. Estimation of number of stem cells per cornea from β-gal positive stripes induced at 12 weeks

(**A, B**) The distribution of LC stripes widths (measured at the cornea-limbal boundary) in eyes of mice injected with tamoxifen at 12 weeks and chased for 6-20 weeks (including eyes 49R, 39L and 39R but excluding eyes 161L and 161R, shown in Supplementary Fig. S1) is shown for all LC stripes (A) and 20-150 µm wide stripes (B). In B one outlier that was < 20 µm and six outliers that were > 150 µm wide were excluded, as the wider stripes are likely to be derived from more than one stem cell. The peak stripe width was 35-40 µm and the overall mean circumference was 10,365 µm, so 259-296 stripes would fit around the limbal circumference. This provides an estimate of the number of stem cells that maintain the corneal epithelium but assumes they are evenly distributed around the circumference. (**C, D**) The distribution of circumference / stripe width ratios at the cornea-limbal boundary (using separate circumference measurements for each eye) is shown for all LC stripes (C) and for 20-150 µm wide stripes (D). The peak circumference / stripe width ratio provides another estimate the number of stripes that would fit around the limbal circumference but the peak estimates were rather broad (175-300). (**E**) Comparison of stripe widths (for 20-150 µm wide stripes) among chase times of 6-20 weeks after tamoxifen injection at 12 weeks by Kruskal-Wallis (KW) tests and Dunn's multiple comparison tests. (Shared letters indicate no significant difference; for other comparisons, $P < 0.05$.) This also tests for differences in stripe widths among

ages because, for this series, age = chase time + 12 weeks. (**F**) Comparison of circumference / stripe width ratios (for 20-150 μm wide stripes) among chase times of 6-20 weeks after tamoxifen injection at 12 weeks by 1-way analysis of variance (ANOVA) and Tukey's multiple comparison tests. (Shared letters indicate no significant difference; for other comparisons, $P < 0.05$.) In the box and whisker plots the whiskers show the minimum and maximum values.

Supplementary Fig S5. Numbers and widths of β-gal positive stripes induced at different ages

(A, B) The number of stripes per cornea was positively correlated between left and right eyes for mice treated with tamoxifen at 4 weeks (A) but not for the smaller sample treated at 24 weeks (B). (For mice treated at 24 weeks, only 13/22 eyes displayed stripes; Fig. 7K.) Pearson's correlation coefficients are shown in the figure. Different chase times and genders are indicated but were not analysed separately. **(C-F)** The distributions of LC stripes widths, measured at the cornea-limbal boundary (C, E) and the circumference / stripe width ratios (D, F) in eyes of mice injected with tamoxifen at 4 weeks and chased for 6 (C, D) or 12 (E, F) weeks. The analysis method is described in the legend to Supplementary Fig S4.

References for Supplementary Figure Legends

Collinson, J. M., Morris, L., Reid, A. I., Ramaesh, T., Keighren, M. A., Flockhart, J. H., Hill, R. E., Tan, S. S., Ramaesh, K., Dhillon, B., West, J. D. (2002). Clonal analysis of patterns of growth, stem cell activity, and cell movement during the development and maintenance of the murine corneal epithelium. Dev Dyn. 224**,** 432-440.

Douvaras, P., Webb, S., Whitaker, D. A., Dorà, N., Hill, R. E., Dorin, J. R., West, J. D. (2012). Rare corneal clones in mice suggest an age-related decrease of stem cell activity and support the limbal epithelial stem cell hypothesis. Stem Cell Res. 8**,** 109-119.

Mort, R. L., Ramaesh, T., Kleinjan, D. A., Morley, S. D., West, J. D. (2009). Mosaic analysis of stem cell function and wound healing in the mouse corneal epithelium. BMC Dev Biol. 9**,** 4.
